# Met Kinetic Signature Derived from the Response to HGF/SF in a Cellular Model Predicts Breast Cancer Patient Survival

**DOI:** 10.1371/journal.pone.0045969

**Published:** 2012-09-25

**Authors:** Gideon Y. Stein, Nir Yosef, Hadar Reichman, Judith Horev, Adi Laser-Azogui, Angelique Berens, James Resau, Eytan Ruppin, Roded Sharan, Ilan Tsarfaty

**Affiliations:** 1 Department of Clinical Microbiology and Immunology, Sackler School of Medicine, Tel Aviv University, Tel Aviv, Israel; 2 Department of Internal Medicine “B”, Beilinson Hospital, Rabin Medical Center, Petah-Tikva, Israel; 3 Blavatnik School of Computer Science, Tel Aviv University, Tel Aviv, Israel; 4 Van Andel Research Institute, Grand Rapids, Michigan, United States of America; University of Nebraska Medical Center, United States of America

## Abstract

To determine the signaling pathways leading from Met activation to metastasis and poor prognosis, we measured the kinetic gene alterations in breast cancer cell lines in response to HGF/SF. Using a network inference tool we analyzed the putative protein-protein interaction pathways leading from Met to these genes and studied their specificity to Met and prognostic potential. We identified a Met kinetic signature consisting of 131 genes. The signature correlates with Met activation and with response to anti-Met therapy (p<0.005) in *in-vitro* models. It also identifies breast cancer patients who are at high risk to develop an aggressive disease in six large published breast cancer patient cohorts (p<0.01, N>1000). Moreover, we have identified novel putative Met pathways, which correlate with Met activity and patient prognosis. This signature may facilitate personalized therapy by identifying patients who will respond to anti-Met therapy. Moreover, this novel approach may be applied for other tyrosine kinases and other malignancies.

## Introduction

Met is the tyrosine kinase receptor (TKR) for Hepatocyte Growth Factor/Scatter Factor (HGF/SF). Met-HGF/SF signaling is crucial for normal development [1]–[5]. Activated Met mutation or Met and/or HGF/SF overexpression are associated with increased angiogenesis, tumorigenesis, invasiveness and metastasis in numerous human solid tumors (www.vai.org/metandcancer) [6], [7]. Overexpression of HGF/SF and Met in breast carcinoma [8]–[10] correlates with triple-negative and basal type tumors [11], [12], and are strong independent predictors of decreased survival [9], [13]–[15], including stage-I patients [16]–[19]. Met overexpression is found in approximately 20% of breast cancer patients [9], [14].

Targeting HGF/SF-Met pathway is becoming an attractive approach for developing anti-cancer agents [20]. The effects of several anti-Met drugs are currently investigated in phase-II and III clinical trials [21]. A crosstalk between Met and other tyrosine kinase signaling have been demonstrated [22]. Only a fraction of the patients respond to targeted therapy and some of those patients ultimately develop resistance, it is therefore necessary to tailor patient specific treatments [23]. Only a handful of cDNA array based Met signatures were published [24]–[26], one of which, a signature based on Met +/− mouse hepatocytes [24], correlates with metastasis and prognosis, but was never validated against large breast cancer patient data sets.

In this work, we generated a distinct Met signature based on kinetic mRNA expression alteration following treatment with HGF/SF on a cellular model. We used Met activation and inhibition cellular and animal models to demonstrate the signatures specificity to Met. Moreover, we have shown the signature’s ability to predict survival in over 1,000 breast cancer patients. Using a protein-protein interaction network analysis tool, we demonstrated the association between Met and its signature genes and identified novel putative Met signaling pathways, which correlate with Met activity as well as with breast cancer patient prognosis.

Our main contributions are: (i) using data derived from a cellular model of TKR activation we identify novel signaling pathways that are specific to the TKR (Met) and correlate with patient survival (ii) we demonstrate the utility of the kinetic signature in determining tyrosine kinase activity *in vivo* and in predicting response to anti-Met therapy in cellular models, potentially serving to personalize anti-Met therapy.

## Results

**Figure 1:**
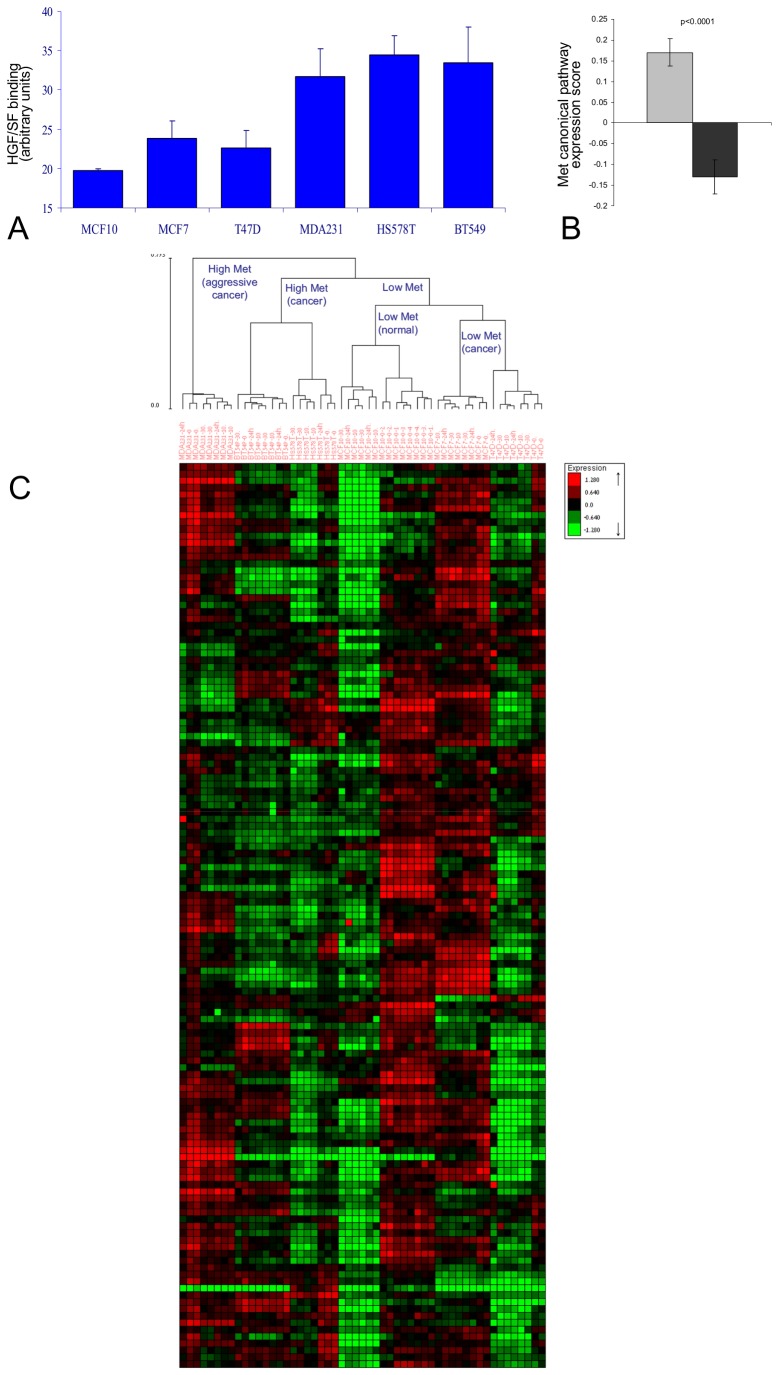
Met signature segmentation of cell line model and human breast cancer patients’ data sets. (A) Cells from six human breast cancer cell lines (MDA231, Hs578T, BT549, MCF10, MCF7 and T47D) were incubated with purified HGF/SF labeled with biotin by a protein biotinylation kit and allowed to bind for 30 min. Cells were then fixed with 4% Paraformaldehyde, permeablized, and stained with Streptavidin-coupled QDot585. Fluorescence levels calculated by image analysis using MICA image analysis software, p<0.0001. (B) Met canonical pathway score calculated by measuring the average mRNA levels of all Met canonical pathway genes (after normalization to average = 0, stdev = 1 per-gene) in high-Met (MDA231, Hs578T and BT549) as compared to the low-Met (MCF10, MCF7 and T47D) samples, p<0.0001. A gray box denotes high Met cell line samples and a black box denotes low Met cell line samples. (C) Hierarchical clustering division of breast cancer cell lines samples using Met kinetic signature genes.

To characterize the effects of Met induction on breast cancer, we studied a cellular model consisting of five human breast cancer cell lines and one normal breast epithelium cell line (MCF10). Three of the cell lines (MDA231, Hs578T and BT549), designated as high-Met, had significantly higher Met levels than the other cell lines (MCF10, MCF7 and T47D) designated as low-Met, as shown by their HGF/SF binding capacity (methods) (p<1e-4, Figure 1A).

To model the kinetic effect of Met activation in low (MCF10, MCF7 and T47D) and high-Met (MDA231, Hs578T and BT549) cell lines, we measured the relative mRNA levels using cDNA array at four different time points (0 min, 10 min, 30 min, and 24 hours following treatment with HGF/SF). These time points represent immediate and late responses to HGF/SF (microarray deposited in ArrayExpress, accession ID: E-MTAB-762, http://www.ebi.ac.uk/arrayexpress/).

Comparing the relative expression of Met canonical pathway genes, Met canonical expression score was found to be significantly higher in the high-Met cell lines as compared to the low-Met cell lines (p<1e-4, Figure 1B). Moreover, hierarchical clustering according to Met canonical pathway genes, perfectly segmented the cell array samples into low and high-Met samples (p<1e-4, Figure S1 A and Text S1). These results demonstrate the Met cellular model specificity to Met.

A Met-related kinetic signature was then constructed by searching for genes that responded to the treatment with HGF/SF and can also differentiate between samples from the high and low-Met cell lines. The genes whose mRNA levels were significantly altered upon HGF/SF treatment (p<1e-4 after FDR correction) were partitioned into six groups according to the time and direction of their initial response (Text S1). The only gene group that gave a perfect separation (p<1e-4) between high and low Met cell lines consisted of 131 genes down regulated after 10 minutes; this group is henceforth referred to as the “Met kinetic signature” (Figure 1 C and Table S1).

Enriched gene ontology (GO) terms and KEGG pathways of Met kinetic signature genes are listed in the Supplementary Results (Text S2).

We further assessed whether the immediate (10 min.) changes in gene expression in response to HGF/SF treatment can be explained by the mRNA half-life of Met kinetic signature genes (Text S1) [27]. We found that indeed Met kinetic signature genes had a significantly shorter mRNA half-life than the rest of the genes in the database (6.99±3.3 vs. 8.9±6.1 hours, p = 0.001). Of note is that the mRNA half life of Met canonical genes did not significantly differ from the rest of the genes in the database (7.7±3.5 vs. 8.9±6.1 hours, p = NS). These results may indicate that the immediate response to HGF/SF on the mRNA level may play an important role in tumorigenicity.

### Met Specificity of Met Kinetic Signature

The ability of the Met kinetic signature to differentiate Met activation/inhibition was assessed in several cellular and animal models. We first used mRNA levels from a transgenic mouse model, expressing an oncogenic variant of Met (Met^mt^), described by Ponzo et al. [12], which spontaneously develops mammary tumors. This analysis demonstrated that hierarchical clustering significantly segmented the samples into a “normal” and “tumor” groups (p<1e-4), thus showing the signatures specificity to Met activation.

Next, we used the Met inhibition cellular model described by Bertotti et al. [26]. In this model, Met-addicted cells were treated with Met inhibitors vs. control. Using hierarchical clustering, Met kinetic signature perfectly separated Met inhibited from the non-inhibited cells (p<0.005).

To demonstrate that the signature does not merely identify high proliferation rates associated with Met activity, we removed all cell-cycle related genes (selected by their GO annotation) from Met kinetic signature and demonstrated that the truncated signature differentiates between: 1) normal and tumor samples in the Met^mt^ transgenic mice [12] and 2) Met inhibited vs. un-inhibited samples [26] (Text S2 and Figure S2).

Taken together, these results may indicate that Met signature may be able to identify Met activation and inhibition in cellular and animal models (Figures S3, S4).

### Molecular Validation of the Met Kinetic Signature

**Figure 2:**
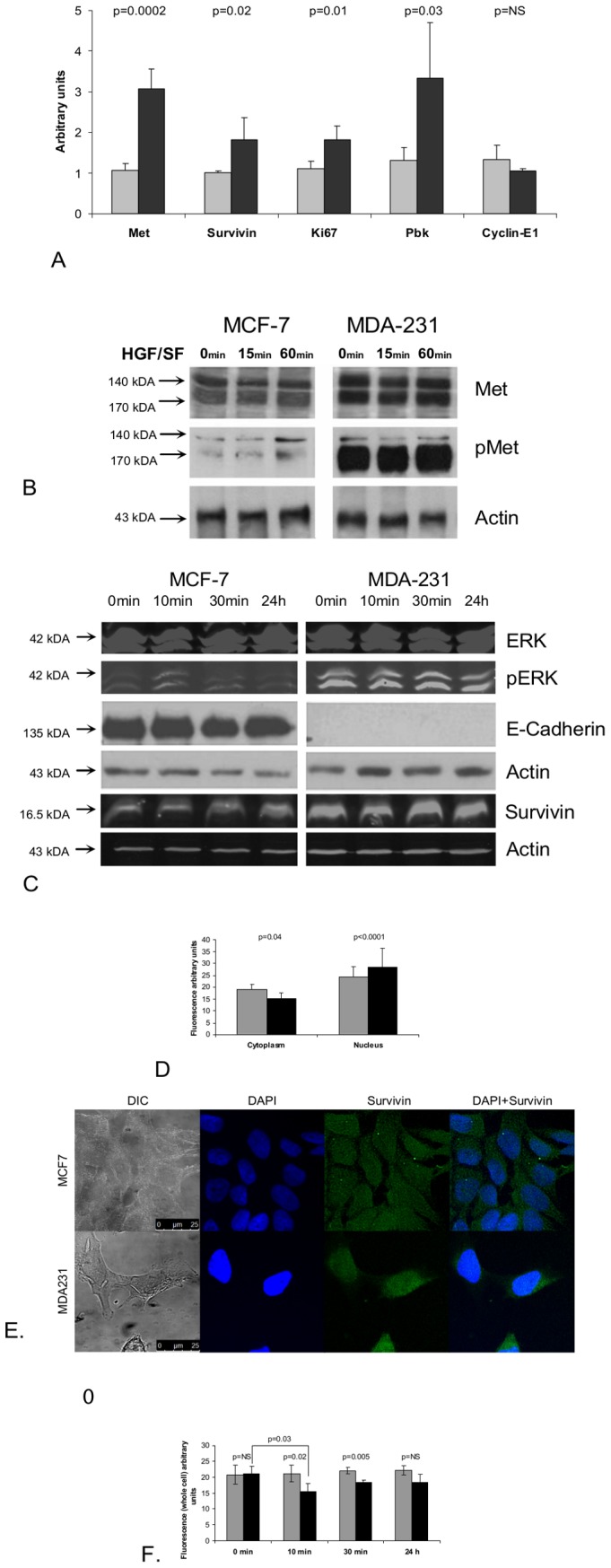
Molecular analysis of Met kinetic signature- mRNA and protein levels of selected genes in high and low Met expressing cells. (A) Total cellular RNA, was isolated from low (MCF7) and high Met (MDA231) cell cultures and mRNA expression of Met, Survivin, Pbk, Cyclin E1 and Ki67 was evaluated by quantitative real time PCR and compared mRNA levels of the housekeeping GAPDH gene. The primers used for the quantification of gene expression are listed in Table S2. A gray box denotes MCF7 cell line samples and a black box denotes MDA231 cell line samples (B) Samples from low (MCF7) and high Met (MDA231) cells were subjected to western blot (WB) analysis, before and 15 min and 60 min after treatment with HGF/SF, using antibodies against Met and activated Met (p-Met) and (C) antibodies against ERK K-23, p-ERK E-4, E-Cadherin, Survivin and Actin C4. (D, E) Subcellular localization of survivin in fluorescence (IF) analysis of Low (MCF7) and high Met (MDA231) cells after treatment with HGF/SF at 0 min, 10 min, 30 min and 24 h. The cells were Immunostained using anti-Survivin antibody. Immunofluorescence was examined using a 510 Meta Zeiss confocal laser scanning microscope (CLSM). Survivin quantification was performed on at least five confocal images per slide. Cell outline was defined based on Nomarski images; nuclei were defined based on the DAPI staining. Average pixel intensity was calculated separately for the nucleus and cytoplasm areas. (F) IF analysis of temporal kinetics of Survivin protein expression following treatment with HGF/SF.

To validate the cDNA array data expression levels, reverse transcription polymerase chain reaction (qRT-PCR) was performed on selected genes from the Met kinetic signature: Survivin, Pbk, Cyclin E1 and Ki67 (The primers used for the quantification of gene expression are listed in Table S2). The results demonstrate that the selected genes analyzed, (except Cyclin E1) are expressed at a higher level in MDA-231 (high Met) as compared to MCF-7 (low Met) cells, in both cDNA microarray and qRT-PCR (Figure 2 A). WB analysis of Met and its phosphorylated form (pMet) and a pivotal Met canonical signaling protein ERK and its phosphorylated form (pERK) demonstrate that Met is expressed at higher levels in MDA231 as compared to MCF7 (1.56 fold) and that Met and ERK are constitutively activated in MDA231 cell line. MCF7 breast cancer cell line expresses low levels of Met and although HGF/SF induced a 1.86 fold increase in Met phosphorylation, its levels are significantly lower than in MDA231 cells, by a factor of 6.4. (Figure 2B, Figure S5). As previously shown E-Cadherin is expressed only in MCF-7 and not in MDA-231 [28]. Survivin levels are 1.9 fold higher in MDA231 as compared to MCF7 (Figure 2 C). Subcellular localization of survivin was detected using immuno-fluorescence (IF) staining. Analysis of IF staining demonstrated that in MDA-231 cells, Survivin is localized in the nucleus as compared to MCF-7 cells, in which Survivin is localized mainly in the cytoplasm (Figure 2 D, F). IF analysis of temporal kinetics of Survivin protein expression following treatment with HGF/SF shows that in MCF-7 cells, Survivin levels are not significantly altered by HGF/SF. However, in MDA-231 cells, Survivin levels are significantly reduced 10 minutes following treatment with HGF/SF (p = 0.03) and then slowly recovered. These results are in accordance with the cDNA array (Figure 2 E and Figure S6).

These results validate the expression levels and activation of Met in the cellular model and the microarray based expression of several Met kinetic signature genes.

### Identifying Novel Met Signaling Pathways

**Figure 3:**
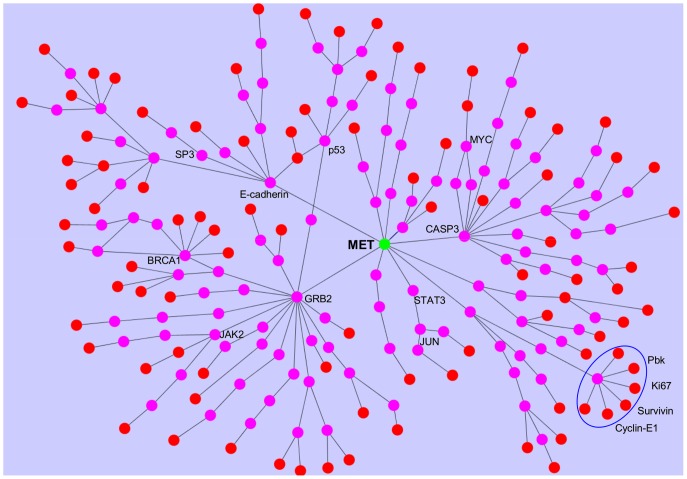
ANAT derived Met anchored network. We used the network inference tool ANAT, to construct the putative protein-protein interaction pathways leading from Met to its kinetic signature genes. ANAT derived Met anchored network is depicted: green nodes– anchor, red nodes - Met kinetic signature genes, pink nodes - nodes selected by ANAT.

Next, we aimed at uncovering the signaling pathways leading from Met to the signature genes using ANAT (Advanced Network Analysis Tool) [29], [30]. The inference is based on projecting Met and the signature genes onto a network of protein-protein interactions (PPI), and searching for a putative compact sub-network that connects them. Overall, ANAT generated 104 pathways, using 95 of the 131 signature genes (36 genes were left out due to insufficient data) (Figure 3). The network model and the list of pathways are provided as Table S3.

Seven of the 32 genes included in the Met canonical pathway are included in the ANAT-derived Met pathways. Moreover, the most common GO annotation is HGF/SF receptor signaling pathway (23%). Other significantly enriched GO annotations are detailed in Text S2.

To validate the ANAT network, we examined if genes that were found to be a part of a specific Met pathway by ANAT are significantly co-regulated (Appendix Methods). We found that the gene-pair correlation distribution comparison of ANAT derived Met network based on Met cellular model is significantly higher than that of all the interacting genes in the ANAT database (p<1e-3) (Figure S7).

To validate the specificity of ANAT’s pathways to Met, we measured the capability of each pathway to differentiate between high and low-Met cell-line samples. We compared the results to a large set of network models derived with randomized data (Methods). The expression score of 38 out of the 104 calculated pathways (36.5%) significantly differentiated high vs. low Met cell lines as compared to a random expectation of 5% (p<1e-4, 30 highly expressed and 8 low/poorly expressed) (Figure S8).

### Prognostic Properties of ANAT Derived Met Network

To assess the prognostic ability of ANAT’s pathways, we measured the capability of each pathway to predict patient survival in three breast cancer patient cohorts (Chang, Miller and van ‘t Veer data sets). We compared the results to a large set of network models derived with randomized data (Methods).

The expression score of 18 (17.3%) pathways correlated with patient survival in all three data sets (p<1e-4). The expression score of 11 of these pathways was significantly higher in high-Met as compared to low-Met samples in the cellular model as well as correlated with poor prognosis in three large breast cancer cohorts (Chang, van ‘t Veer and Miller, Table 2). Interestingly, the expression of pathways whose genes have similar kinetic response to HGF/SF (pathway with high coherency ranking) in the cellular model, correlate with poor patient outcome (spearman rank correlation was -0.316 with p = 0.001). These results indicate that the coherent pathways play a critical role in Met signaling, affecting tumorigenesis and metastasis.

To further validate the specificity of ANAT network to Met, we used ANAT to compute the pathways leading from four alternative different tyrosine kinases serving as anchors (EGFR, ERbB2, INSR and PDGFRA) to the 131 genes composing the Met kinetic signature. Met and ERbB2 ANAT derived network showed similar number of prognostic pathways (17.3% and 16.6%, respectively, p = NS). However, significantly more Met pathways (17.3%) correlated with patient prognosis, as compared to the EGFR, ERbB2, INSR and PDGFRA derived ANAT pathways respectively (7.1%, 6.4% and 5.4%, respectively, p<0.01).

These results demonstrate the prognostic value of the Met anchored ANAT derived pathways, the similarity between Met and ERbB2 signaling pathways and it’s specificity as compared to the other pathways.

### Prognostic Properties of Met Kinetic Signature

mRNA levels and clinical data from three large human breast cancer patient data sets (Materials and Methods) [31]–[33] were used to evaluate the prognostic ability of Met mRNA levels, of its canonical pathway levels and of the Met kinetic signature. In contrast to Met protein levels [8], [9], the mRNA levels of Met alone and those of the Met canonical pathway genes did not correlate with patient survival in all three data sets (Figure S1 B, C).

**Figure 4:**
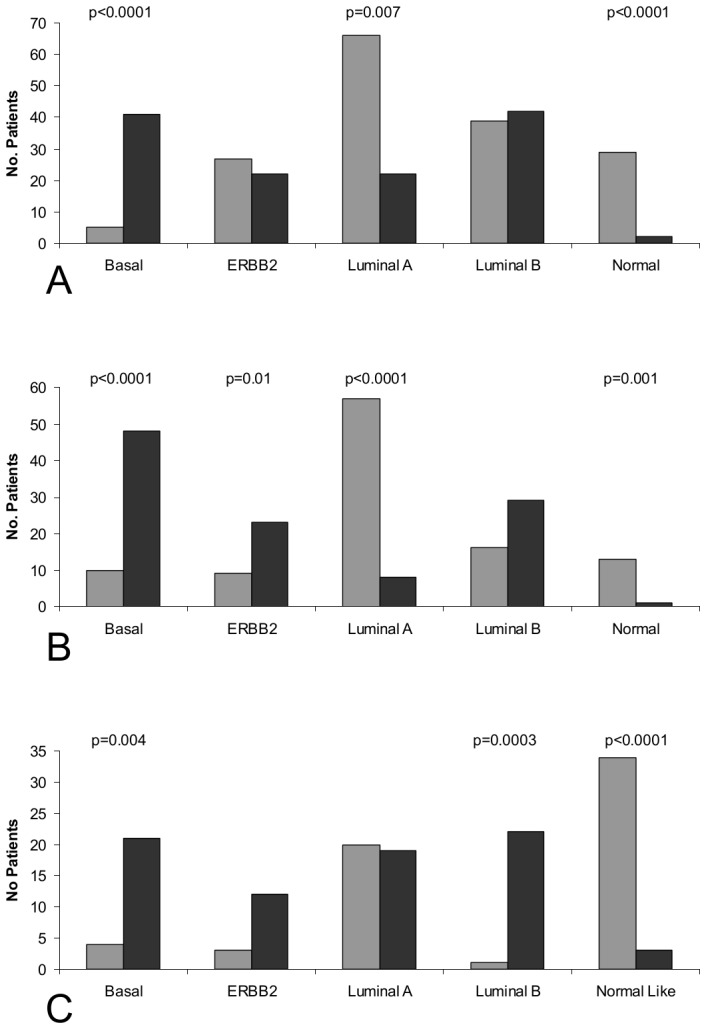
Analysis of the association between High Met kinetic signature and basal-like tumors. Hierarchical clustering was used to divide three large breast cancer patient cohorts (Chang (A), GSE3165 (B) and GSE1456 (C)), according to Met kinetic signature genes. The resultant patient groups were analyzed for association with tumor molecular classification. A gray box denotes patients in the low Met activity group and a black box denotes patients in the high Met activity group.

**Figure 5:**
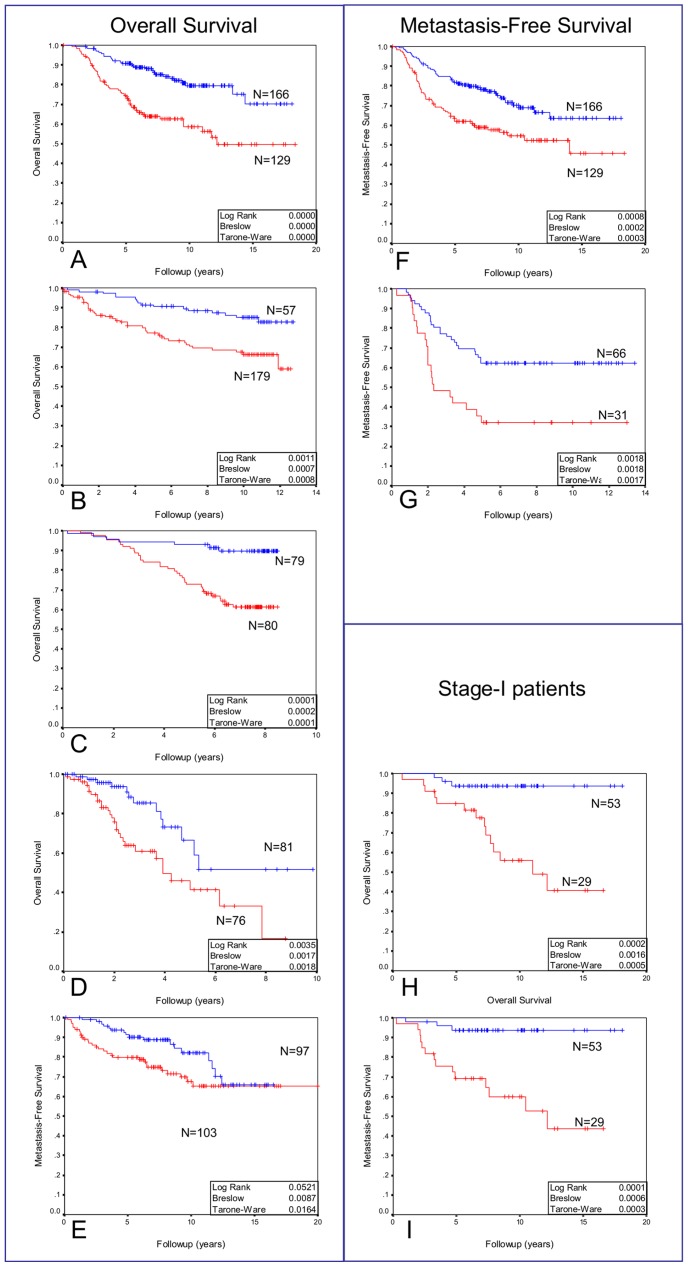
Kaplan Meier survival analysis of Met kinetic signature’s segmentation of human breast cancer patient cohorts. Hierarchical clustering was used to divide six large breast cancer patient cohorts into high vs. low Met kinetic signature. Kaplan Meier analysis of overall survival (A,B,C,D,E) and metastasis-free survival (F,G) of the Chang (A, F, H, I), Miller (B), GSE1456 (C), GSE3165 (D), GSE11121 (E) and van ‘t veer (G) data sets. Kaplan Meier analysis of overall survival (H) and metastasis-free survival (I) of stage-I patients in the Chang data set. A red line denotes patients with high Met kinetic signature and a blue line denotes patients with low Met kinetic signature. In Chang data set, Met kinetic signature has a positive predictive value (PPV) and negative predictive value (NPV) of 41% and 82%, respectively.

Turning next to test the Met kinetic signature as differentiator criteria between different patient characteristics, we used the signatures genes to cluster the patients in each of six large human breast cancer patient data sets [31]–[36] into two groups. The average Met canonical expression levels in each of the resulting groups revealed a classification into either “High Met kinetic signature” or “Low Met kinetic signature” (Table S4). We found that the High Met kinetic signature is significantly associated with basal tumors (p<1e-3), and that the low Met kinetic signature is significantly associated with normal-like and luminal-like tumors (p<1e-3) in the Chang, GSE3165 and GSE1456 data sets (Figure 4). Additionally, the high Met kinetic signature correlated with low estrogen and progesterone receptors, p53 mutations, high histological grade, BRCA1 mutations, triple-negative tumors and metastasis (Table 1). Finally, a Kaplan Meier analysis showed that patients in the high Met kinetic signature groups have significantly reduced metastasis-free and overall long term survival in all six cohorts (n = 1145 p<0.01). Moreover, the high Met kinetic signature levels correlated with reduced metastasis-free and overall survival in patients with stage I disease (Chang data set) (p<0.01) (Figure 5).

**Table 1:** Met Kinetic Signature correlation with clinical and histopathological data.

	Miller	Van ‘t Veer	Chang	GSE3165	GSE1456	GSE11121
Young age	NS	0.006	NS	0.04		
Size	0.001	0.02	0.003	NS		0.05
Node status	NS		NS	NS		
Metastasis		0.006	0.003			
BRCA1 mutation		<0.0001				
p53 mutation	<0.0001					
Estrogen receptor (−)	0.02	<0.0001	<0.0001	<0.0001		
Progesterone receptor (−)	0.007	<0.0001		<0.0001		
High histological Grade	<0.0001	<0.0001	<0.0001	0.0007	<0.0001	<0.0001
Lymphocytic Infiltrate		<0.0001				
Basal-like tumors			<0.0001	<0.0001	0.004	
Triple negative tumors				<0.0001		
Reduced metastasis-Free Survival		0.0018	0.0008			
Reduced overall Survival	0.001		<0.0001	0.003	0.0001	0.01
Reduced stage I - Survival			0.0001			

Comparing the prognostic value of the signature in predicting patient survival to 100 random signatures, the original signature outperformed all the randomly chosen ones, testifying to its validity (Methods).

We also found that Met kinetic signature correlated with patient survival independent of Basal-like classification (in 3 of 3 cohorts, p<0.05), estrogen receptor (ER) status (in 2 of 4 cohorts, p<0.05) and histological grade (in 3 of 6 cohorts, p<0.05). Moreover, the Met kinetic signature correlated with patient survival even after removal of cell-cycle genes (in 5 of 6 cohorts, p<0.05) and is comparable to the 70 gene signature [37] (Text S2 and Table S5 and Figure S9).

Thus Met kinetic signature is associated with triple negative basal like tumors, and identifies patients with reduced metastasis-free, disease-related and overall survival.

These results suggest that Met kinetic signature may serve to identify breast cancer patients with activated Met pathways and high risk of an aggressive disease, who would benefit from personalized anti-Met therapy.

## Discussion

In the past decade, several microarray-based prognostic signatures were developed, that combined with other traditional clinical factors, facilitate management of cancer. Currently, however, cDNA based signatures do not predict response to targeted therapies. In this work we lay the grounds for the identification of breast cancer patients most likely to benefit from anti-Met therapy.

We have used, a cellular model which simulates a specific TKR activation to generate a molecular signature which is then applied on other cellular and animal models combined with several large scale patient cohorts, strongly supporting its validity. Moreover, the signature relation to the TKR was validated by activation and inhibition models as well as by protein-protein interaction network analysis. We postulate that this methodology may be used to reveal new signaling pathways and predict response for other oncogenes.

Careful design of the cellular model is critical for its ability to best differentiate between tumors with low and high Met activity. To this aim we: 1) used breast cancer cell lines with low and high Met expression and activation; 2) used several cell lines in each group to highlight Met activation features which are not cell line specific; 3) measured short and intermediate temporal changes in gene expression following Met activation by HGF/SF and 4) used multiple replications to ensure data integrity. Taken together, these measures ensured our cellular model identifies differences in Met activation between low and high Met samples with high specificity.

Although the cells were selected only based on their Met expression levels and their response to HGF/SF treatment, the high-Met cell lines were assigned to the Claudin-low subtype by the Perou group while the low-Met cell lines are either of the luminal cancer subtype or normal cells (MCF10) [38]. The association between Met activation and basal-like tumors is well documented [11], [12], [39], [40] and it is therefore understandable that high-Met expressing cell lines are assigned to the Claudin-low subtype group while the Met-low cell lines are either of the luminal cancer subtype or normal cells.

We started by generating Met kinetic signature, based on the kinetic response to HGF/SF of cell lines with high or low Met expression. The fact that Met kinetic signature classified the cell lines to Met-level groups indicates that these genes play a major role in regulating the biological activities induced by Met.

We do not fully understand why genes which expression is down regulated shortly after HGF/SF treatment, on one hand differentiate between High and Low Met samples in the cellular model, and on the other hand high expression of these genes correlate with poor prognosis. One possible explanation relates to the role of many of these gene products in cell cycle regulation and DNA replication and repair. Such genes’ expression is tightly regulated with regard to the cell cycle phase. In the cellular mode, the kinetics measured hint towards short-term HGF/SF induced cell cycle synchronization as opposed to the altered expression in the unsynchronized tumor cells. These immediate changes in the expression of Met kinetic signature genes may also relate to the relatively short half life of Met kinetic signature genes. Moreover, the immediate response might be more specific to Met while the long term response may represent the similarity in all TKR signaling pathways.

The classification between High and Low Met kinetic signature correlates with histopathological and clinical features. Thus, most of the tumors in the “High Met kinetic signature” group were basal-like tumors and the patients had reduced disease free intervals and poor prognosis independent of its Claudin classification (Table S5). These results are in accordance with the results published recently, which show that Met-related tumors are basal-like and are associated with poor outcome [11], [12].

To assess the relation between Met and its kinetic signature genes, we used ANAT, a new interactive software tool for elucidating functional networks of proteins [29], to map the pathways leading from Met to its signature genes. We have shown that the ANAT derived Met network is specific to Met by showing that: 1) the proportion of ANAT derived pathways who are enriched with HGF/SF receptor signaling pathway is significantly high; 2) the gene-pair correlation distribution comparison of ANAT derived Met network based on Met cellular model is significantly higher than that of all the interacting genes in the ANAT database and 3) the proportion of ANAT derived pathways which differentiate between high vs. low Met cell lines samples is significantly high.

We have then showed that the ANAT derived Met network is enriched with pathways whose gene values correlate with patient prognosis. Moreover, networks recalculated the after replacing Met with EGFR, ERbB2, INSR and PDGFRA, yielded far less prognostic pathways, indicating the signatures specificity to Met and patient prognosis.

The ANAT derived network linking Met and its kinetic signature genes revealed eleven novel putative pathways which correlate with Met activity and breast cancer patient prognosis. These pathways contain genes that are involved in cell migration, invasion, proliferation, cell cycle and some correlate with anti-estrogen resistance and p53 deactivation. Genes from all eleven pathways were found to be associated with Met and with cancer progression (Table 2). Detailed analysis on 5 of these pathways and their relation to Met activity and breast cancer can be found in Text S2.

**Table 2:** ANAT derived pathways that correlate with Met activity and prognosis.

ANAT Derived Pathways	p-value (Metcellular model)	p-value (prognosis on3 BC cohorts)	Association with Met	Association with cancer
MET - CASP3 - CDKN1A - DTL	0.0012	0.0067	[48], [49]	[48], [50]
MET - CASP3 - PARP1 - POLA2	0.0013	0.0038	[48], [49]	[48]
MET - CBL - LYN - CDC2 - Survivin	<0.0001	0.0022	[40], [51]–[54]	[40], [55]–[58]
MET - CBL - LYN - CDC2 - CCNE1	0.0090	0.0052	[40], [51], [52], [54]	[40], [57]–[59]
MET - CBL - LYN - CDC2 - MKI67	<0.0001	0.0220	[17], [40], [51], [52], [54]	[40], [57], [58]
MET - CBL - LYN - CDC2 - PBK	<0.0001	0.0072	[40], [51], [52], [54]	[40], [57], [58], [60], [61]
MET - CDH1 - RRM2	0.0003	0.0052	[62], [63]	[63], [64]
MET - GRB2 - BCAR1 - YWHAZ - MLF1 - MLF1IP	0.0070	<0.0001	[65]	[66]–[68]
MET - GRB2 - JAK2 - STAM - STAMBP - CTNNBL1	0.0002	0.0390	[69], [70]	[71], [72]
MET - GRB2 - PTK2 - TP53 - NP - TFAM	0.0079	0.0318	[19], [73], [74]	[73]–[75]
MET - GRB2 - PTK2 - TP53 - RRM2 - RRM1	0.0003	0.0148	[19], [73], [74]	[73], [74], [76]

p-values for differentiation between high and low Met samples in the cellular model and for differentiation between patients with good and poor prognosis in three large BC patient cohorts (Chang, Miller and van ‘t Veer) are provided. Reference for the association between the pathway genes, Met acivity and cancer progression are also provided.

Importantly, we found a correlation between the coherencies of the ANAT derived Met pathways in the cellular model and their prognostic value in breast cancer patients, indicating biological co-regulation of prognosis-associated Met pathway genes.

Multiple analyses were used to demonstrate the signature’s specificity to Met and its robustness as prognostic marker for breast cancer. Specifically, by demonstrating its prognostic capabilities on more than 1000 patients in six different breast cancer patient cohorts.

To establish its role in identifying breast cancer patients who will most benefit from anti-Met therapy, we showed that Met kinetic signature: 1) identifies high Met activity in a cellular model as well as Met-specific tumors in a Met^mt^ animal model; 2) differentiates Met inhibited cells from control in a Met-addicted cellular model and 3) predicts long term prognosis on a large cohort of patients. Met pathway signature was found to be comparable to other classification methodologies (i.e. the 70 gene signature [37] see Text S2). In contrast to the 21-gene signature, Oncotype DX [41], which predicts prognosis only in ER-positive, node-negative disease, our signature predict outcome in ER negative and positive patients in different stages of the disease.

Met kinetic signature may also open a new spectrum of therapy targets, through understanding of the molecular mechanism of Met activity in the tumor. We hypothesize that this signature will enable personalized therapy by identifying patients in which anti-Met therapy will suppress Met downstream signaling and delay tumor progression. Clinical trials are needed to study Met pathway signature’s role as a clinical tool to identify breast cancer patients most likely to benefit from personalized anti-Met therapy.

## Methods

Details regarding the preparation and characterization of the cellular model, generation of the cDNA arrays its validation by qRT-PCR, immunofluorescence and Western blot, as well as description of Met kinetic signature generation are listed in Text S1.

### Cell Culture

Human breast cancer cell lines expressing low Met (T47D, MCF7),or high constitutively activated Met (Hs578T, BT549, MDA231) and a human cell line derived from normal breast epithelium (MCF10 [42]) (obtained from American Type Culture Collection, Bethesda, MD), were cultured in 1640 RPMI medium containing 5% FBS, and PSN (Biological Industries, Israel). Hs578T, BT549, MDA231 and MCF10 are estrogen receptor (ER) negative while T47D and MCF7 are ER positive [43], [44].

### HGF/SF Binding

Breast carcinoma cells were grown on glass bottom plates (MatTek, MA, USA). Cells were incubated with purified HGF/SF [45] labeled with biotin by a protein biotinylation kit (Amersham Biosciences) and allowed to bind for 30 min. Cells were then fixed with 4% Paraformaldehyde, permeablized, and stained with Streptavidin-coupled QDot585 (Quantum Dot Corporation, Carlsbad California USA). Fluorescence levels were analyzed using MICA (CytoView Ltd, Petah-Tikva, Israel).

### Published Human Breast Cancer Microarray Data Sets

We used six published large data sets of genome-wide expression measurements taken from breast cancer patients: (i) The Miller data [32] consists of 251 primary breast tumors, clinical indices include overall patient survival. (ii) The Van’t veer data [31] set is based on biopsies from 117 young, node-negative breast cancer patients. Clinical indices include BRCA1 mutation status and metastasis-free survival. (iii) The Chang data [33] is based on a consecutive series of 295 early breast cancer patients which was used to validate a “wound-response” signature. Notably, there is an overlap of 31 patients between Van’t veer and Chang data sets. Clinical indices include metastasis-free and overall patient survival. (iv) GSE3165 data [34] is based on 232 human breast tumor and normal tissue arrays, which was used to validate a Met^mut^ mammary tumor model, described by Graveel et al [11]. (v) GSE1456 data [35] is based on 159 breast cancer samples. (vi) GSE11121 data set [36] is based on 200 tumors of breast cancer patients who were not treated by systemic therapy after surgery.

### Calculating a Whole-pathway Expression Level

To assess the expression level of a given pathway in a given sample, we calculated the average z-score of all the genes in the pathway. We used this procedure to define the expression of the Met canonical pathway (the *Met canonical expression score*), including a list of Met canonical pathway genes according to published literature (Text S1) [6].

We calculated the expression score of pathways derived from the protein-protein interaction (PPI) network (described below) in the same manner.

### Evaluating the Prognostic Value of Met Kinetic Signature on Human Breast Cancer Patients

We evaluated the predictive power of the signature with respect to several phenotypes (i.e., high vs. low Met cell-lines, patient survival, lymph-node status, tumor classification etc.) by clustering the patients into two groups using hierarchical clustering as previously described.

Met canonical expression score was then calculated for patients in each group. The group with the higher score average was designated “High Met kinetic signature” and the other group was designated “Low Met kinetic signature” (the differences between the group are depicted in Table S4). We then tested the concordance between the two groups and several clinical phenotypes. For the survival phenotypes we used a Kaplan-Meier analysis. For discrete phenotypes such as BRCA1 mutation status, we used a Chi-square test. For continuous phenotypes (age, and tumor size), we used two-tailed t-test, comparing the distribution of phenotype levels in the two groups.

To study the specificity of the prognosis obtained using the kinetic signature, we compared the p-values obtained from the Kaplan-Meier analysis to those obtained with 100 random signatures (of similar size as the signature) sampled from the cDNA array. We evaluated the prognostic value of each signature by taking a lower bound on its performance across all three (Chang, Miller and Van’t Veer) data sets (i.e., taking the maximum p-value). To further validate Met kinetic signature, we evaluated its association with classical (TNS-tumor grade, number nodes, stage) tumor characteristics and patient prognosis, on additional three human breast cancer data sets [34]–[36].

### Constructing a Protein-protein Interaction Network Model

ANAT (Advanced Network Analysis Tool) is a new interactive software tool for elucidating functional networks of proteins [29]. It encompasses a number of state-of-the-art network inference algorithms and provides access to up to date networks of experimentally validated PPIs in several organisms (including human). Every interaction in the database is assigned with a confidence score based on the number and type of experiments in which it was observed. In difference from existing software tools, ANAT is uniquely capable of inferring network models that connect hundreds of proteins to each other or to a given set of “anchor” proteins.

We used ANAT to construct the most likely network that connects the proteins encoded by the newly found signature to Met (acting as an anchor). After an initial construction of the network, we performed minor adjustments by forcing directionality on several of the edges (from HGF/SF to Met, from GRB2 to EGFR and from CBL to EGFR), and by removing FGFR1 and FGF3 (as they are in parallel to Met pathway and not part of it). If a specific pathway was found to be included in another, the longer pathway was excluded from the network.

### Evaluating the Specificity of the ANAT Derived Met Pathways and their Prognostic Value

The specificity of the ANAT predicted Met pathways’ was assessed by comparing each pathway’s expression score in high versus low-Met cell lines. To evaluate its prognostic value in breast cancer patients, we compared the expression score of each pathway between patients with good prognosis (at least 5 year follow-up, who did not die from the disease or developed metastasis during follow-up) and patients with bad prognosis (those who died from the disease or developed metastasis at any time during follow-up). On both tests, significance is estimated using a two-tailed t-test.

To evaluate the specificity of the results, we performed two analyses using (i) another tyrosine kinase growth factor receptor as an anchor to the Met signature genes and (ii) using ANAT to construct 100 Met anchored networks using 100 random signatures of the same size as the Met kinetic signature. We compared the significance levels obtained for the pathways of the original network to those of the random pathways, determining a pathway as significant if its level scored higher than 95% of the random pathways. To evaluate the significance of the Gene Ontology (GO) [46] annotation enrichment of the Met-signature pathway genes, we compared to random pathways genes GO annotation using a hypergeometric test.

To further validate the ANAT network, we examined if genes that were found to be a part of a specific Met pathway by ANAT are significantly co-regulated. Indeed, it was recently shown that two proteins that share the same pathway and are co-regulated by specific factors/elements are also co-expressed and their expression levels are correlated [47]. To perform this test, we calculated the correlations of mRNA levels (in the 6 cell-lines data) between genes that are connected by an edge in the ANAT derived network. As a background control we computed the correlations between all the interacting genes in the ANAT database (over 40,000 pairs). Comparison of the two distributions was performed using Wilcoxon-Mann–Whitney test and *t*-test.

### Cellular Model Derived Met Pathway Coherency and its Relation to Patient Survival

The coherency ranking of a pathway is defined as the average correlation of consecutive pairs of genes along the pathway. For each pathway we calculated: 1) the coherency ranking of the ANAT derived pathways based on the cellular model (after normalization to average = 0, stdev = 1 per-gene, per-cell line), 2) pathway expression score for each patient in the breast cancer patient data sets. We calculated each pathway’s predictive value by comparing the pathway expression score between good and bad prognosis patients using student’s t-test on each data set. The log_10_() of the worst p-value between all data sets was designated as the pathway’s prognosis ranking. The relation between the coherency and the prognosis ranking of ANAT derived pathways was determined using Spearman’s rank correlation.

## Supporting Information

Figure S1 **Hierarchical clustering of the cell line model according to Met canonical pathway genes.** Hierarchical clustering of the breast cancer cell line model according to Met canonical pathway genes, perfectly segmented the cell array samples into low and high-Met samples (p<1e-4) (A). Met canonical pathway score correlates with patient survival in only one of three breast cancer patient data sets (B). Met mRNA levels did not correlate with patient survival in all three breast cancer patient data sets (C).
(PDF)

Figure S2 **Met kinetic signature after removal of cell cycle genes, identifies Met activity and predicts survival.** Cell cycle genes (according to their GO annotation) were removed from the Met kinetic signature, resulting in a 96 gene signature. The reduced signature significantly correlated with Met activation animal model (A) and Met inhibition cellular model (B) and predicted survival in five of six large breast cancer patient cohorts: van ‘t Veer (C), Miller (D), Chang (E), GSE3165 (F), GSE1456 (G) and GSE11121 (H).
(PDF)

Figure S3 **Hierarchical clustering of mutationally activated Met mouse model according to Met kinetic signature genes.** We used mRNA levels from a mutationally activated Met mouse model and found that using Met kinetic signature, hierarchical clustering significantly segmented the samples into a “normal” and “tumor” groups (p<1e-4).
(PDF)

Figure S4 **Hierarchical clustering of Met inhibition cellular model according to Met kinetic signature genes.** Using the Met inhibition cellular model described by Bertotti et al., we found that Met kinetic signature perfectly separated Met inhibited samples in the high-Met cell line and also the EGFR inhibited samples in the EGFR-addicted samples (p<0.005).
(PDF)

Figure S5 **Quantification of Western blot analysis.** Basal levels of Met are 1.56 times higher in MDA231 as compared to MCF7 cells and treatment with HGF/SF did not significantly change Met basal levels in either both cell lines (A). Levels of pMet are 6.4 times higher in MDA231 as compared to MCF7 cells. Sixty minutes following treatment with HGF/SF, pMet levels are 1.86 higher in MCF7 as compared to base line, but are still significantly lower than in MDA231, whose pMet levels did not significantly change following treatment with HGF/SF (B). Levels of ERK in MCF7 and MDA231 are similar (C). Levels of pERK are 15 times higher in MDA231 as compared to MCF7 cells. Levels of pERK did not significantly change following treatment with HGF/SF (D). As expected, E-cadherin levels are almost undetected in MCF7 cells and in MDA231, its levels are elevated following treatment with HGF/SF (E). Survivin levels are 1.9 higher in MDA231 as compared to MCF7 cells and do not significantly change following treatment with HGF/SF (F).
(PDF)

Figure S6 **Survivin immuno-fluorescence in MCF7 and MDA231 cells.** MCF7 and MDA231 cells were incubated with the primary antibody anti-Survivin (Santa Cruz, 1∶50). Slides were analyzed using a 510 Meta Zeiss confocal laser scanning microscope (CLSM). When comparing fluorescence intensities, identical CLSM parameters (e.g. pin hole, scanning line, laser light, contrast and brightness) were used. To compare the relative levels of protein expression, we used the average area intensity (AAI) image analysis procedure for cells immunostaining. The image analysis calculations were performed on five to ten microscopic fields. Cell outline was drawn based on DIC images; nuclei were defined based on the DAPI staining. Average pixel intensity was calculated separately for the nucleus and cytoplasm areas. (MICA software; Cytoview, Petach Tikva, Israel). Variance was analyzed by student’s T-test.
(PDF)

Figure S7 **Gene-pair correlation distribution comparison of ANAT derived Met network.** The gene-pair correlation distribution comparison of ANAT derived Met network is significantly higher then that of all the interacting genes in the ANAT database (p = 0.0006).
(PDF)

Figure S8 **Expression score of ANAT-derived pathways.** The expression score of 38 out of the 104 calculated pathways (36.5%) significantly differentiated High vs. Low Met cell lines as compared to a random expectation of 5% (p<1e-4, 30 highly expressed and 8 low/poorly expressed).
(PDF)

Figure S9 **Subgroup analysis of Met kinetic signature by ER status.** Subgroup analysis of Met kinetic signature by ER status in van ‘t Veer, Miller, Chang and GSE3165 data sets showed that high-Met kinetic signature correlated with poor prognosis in ER+ patients, but not in ER- patients.
(PDF)

Table S1 **Met Kinetic Signature genes.**
(PDF)

Table S2 **Primers for qRT-PCR. The following primers are used for the quantification of gene expression.**
(PDF)

Table S3 **ANAT derived pathways originating from Met to the kinetic signature genes.**
(PDF)

Table S4 **Differences in Met canonical pathway expression score between patient groups segmented by Met kinetic signature.**
(PDF)

Table S5 **Cox proportional hazards regression survival analysis using Met kinetic signature and basal-like classification on three breast cancer patient cohorts.**
(PDF)

Text S1 **Supplemental Methods.**
(DOC)

Text S2 **Supplemental Results.**
(DOC)
