# Supplementary figures and images for: Met Kinetic Signature Derived from the Response to HGF/SF in a Cellular Model Predicts Breast Cancer Patient Survival
Source: PLoS One. 2012 Sep 25;7(9):e45969. doi: 10.1371/journal.pone.0045969 (PMC3457970; doi:10.1371/journal.pone.0045969)

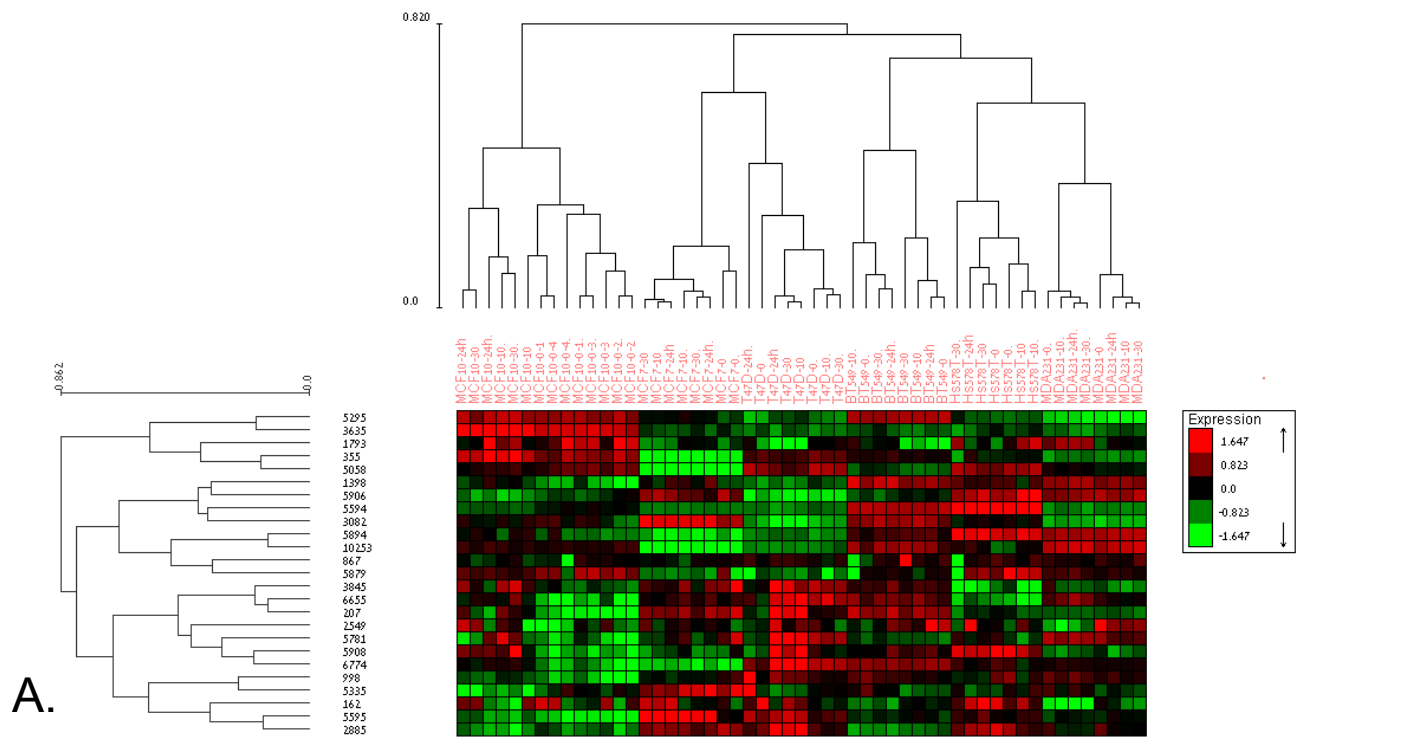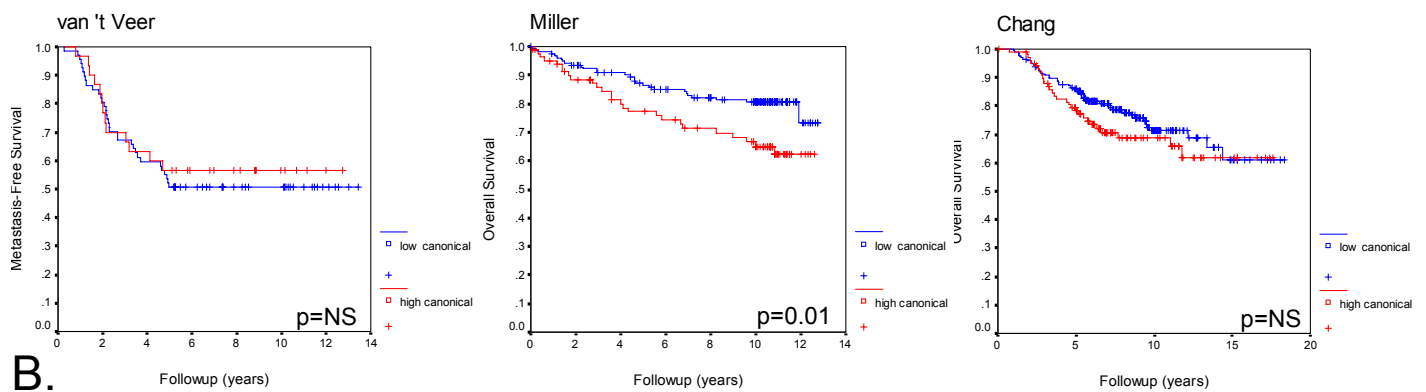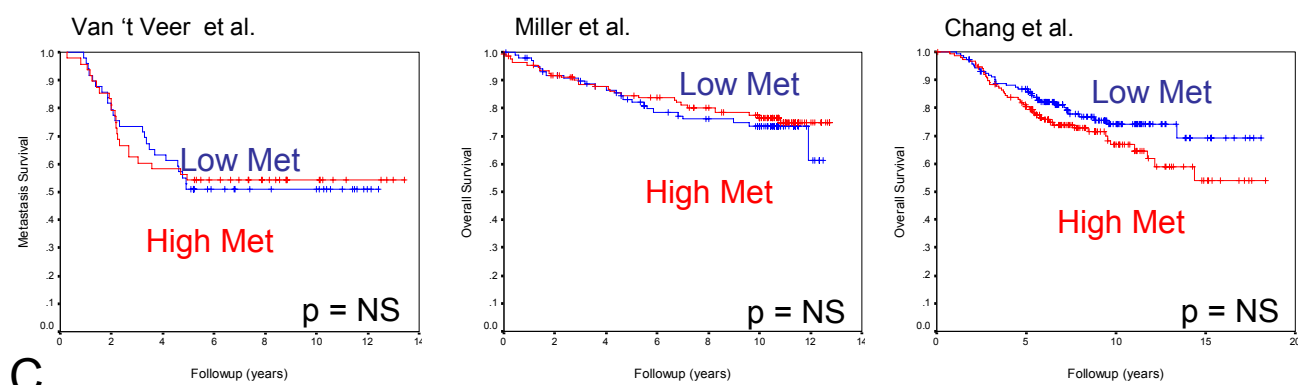

Supplement: Figure S1 — Hierarchical clustering of the cell line model according to Met canonical pathway genes. Hierarchical clustering of the breast cancer cell line model according to Met canonical pathway genes, perfectly segmented the cell array samples into low and high-Met samples (p<1e-4) (A). Met canonical pathway score correlates with patient survival in only one of three breast cancer patient data sets (B). Met mRNA levels did not correlate with patient survival in all three breast cancer patient data sets (C). (PDF) [file pone.0045969.s001.pdf]

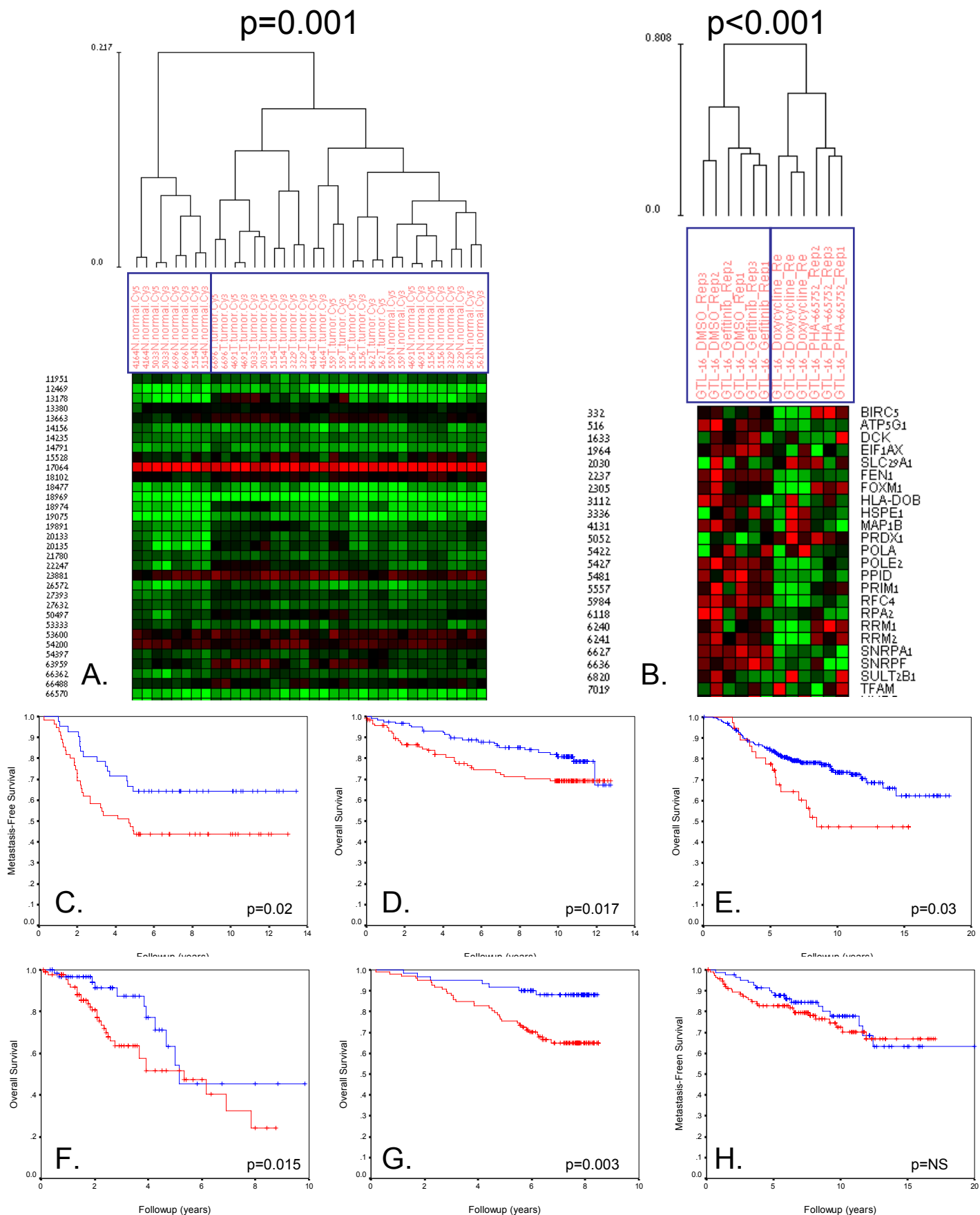

Supplement: Figure S2 — Met kinetic signature after removal of cell cycle genes, identifies Met activity and predicts survival. Cell cycle genes (according to their GO annotation) were removed from the Met kinetic signature, resulting in a 96 gene signature. The reduced signature significantly correlated with Met activation animal model (A) and Met inhibition cellular model (B) and predicted survival in five of six large breast cancer patient cohorts: van ‘t Veer (C), Miller (D), Chang (E), GSE3165 (F), GSE1456 (G) and GSE11121 (H). (PDF) [file pone.0045969.s002.pdf]

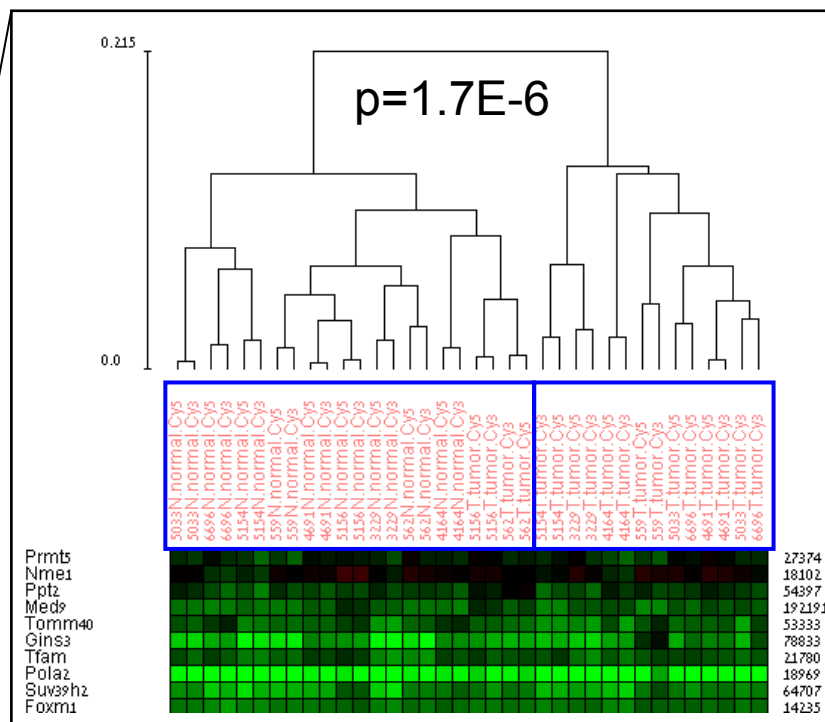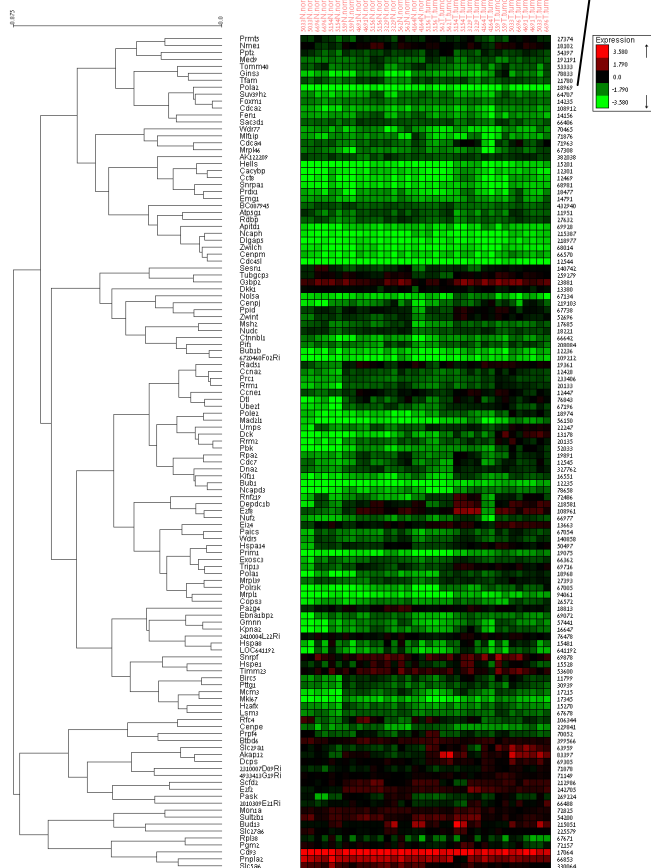

Supplement: Figure S3 — Hierarchical clustering of mutationally activated Met mouse model according to Met kinetic signature genes. We used mRNA levels from a mutationally activated Met mouse model and found that using Met kinetic signature, hierarchical clustering significantly segmented the samples into a “normal” and “tumor” groups (p<1e-4). (PDF) [file pone.0045969.s003.pdf]

GTL - High-Met, Low-EGF  
DiFi - Low-Met, High-EGF

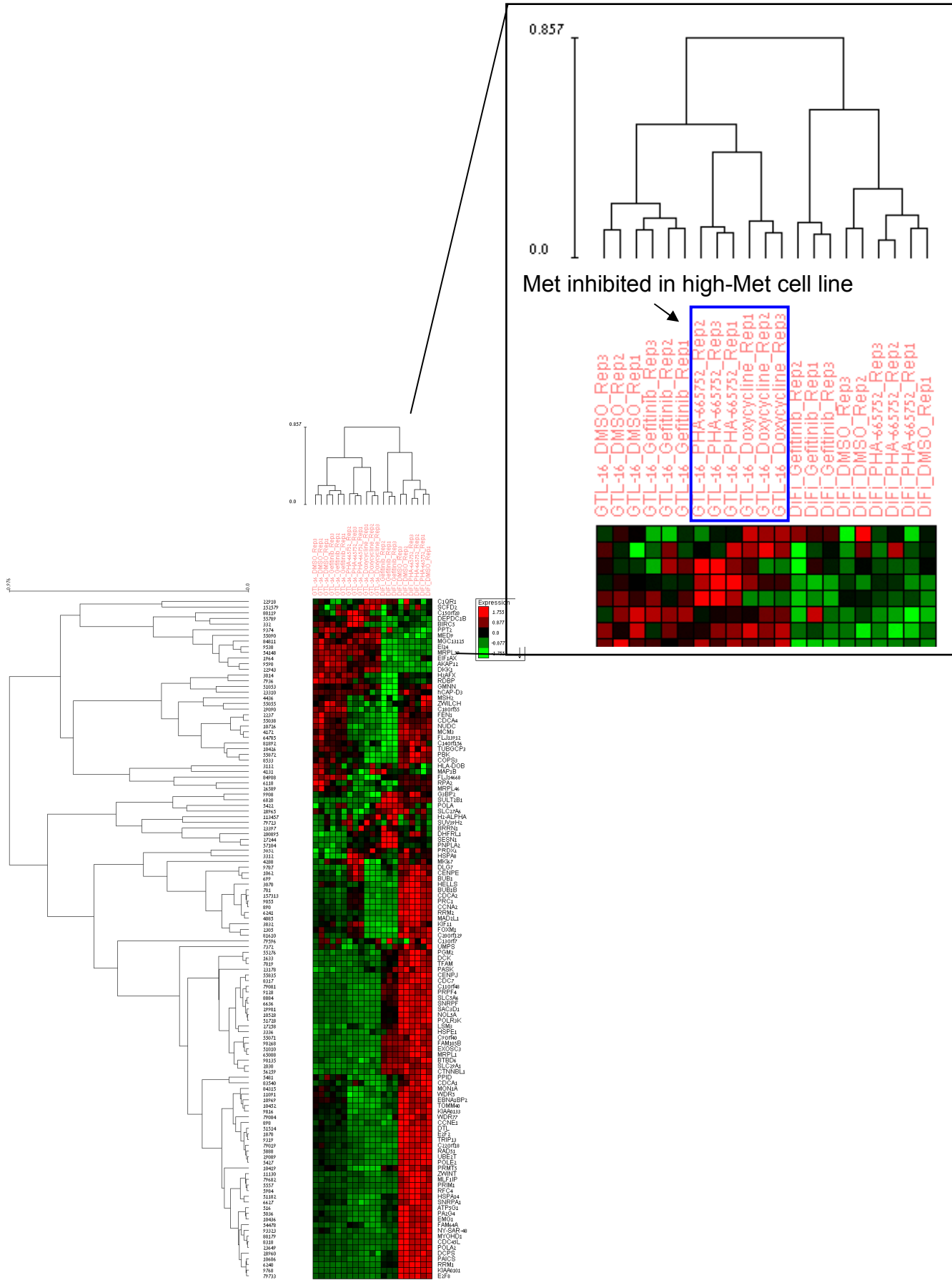

Supplement: Figure S4 — Hierarchical clustering of Met inhibition cellular model according to Met kinetic signature genes. Using the Met inhibition cellular model described by Bertotti et al., we found that Met kinetic signature perfectly separated Met inhibited samples in the high-Met cell line and also the EGFR inhibited samples in the EGFR-addicted samples (p<0.005). (PDF) [file pone.0045969.s004.pdf]

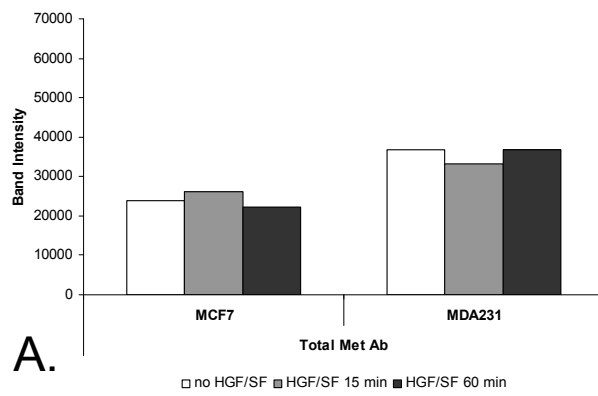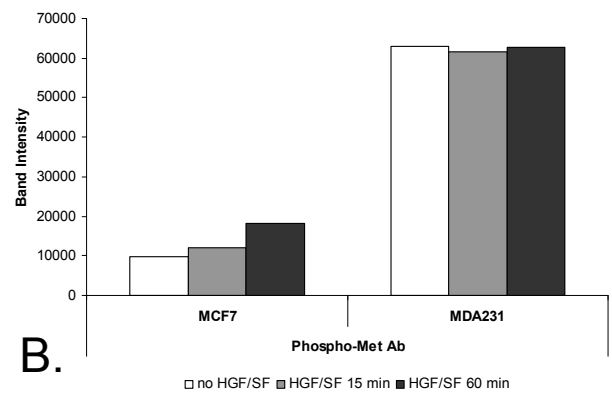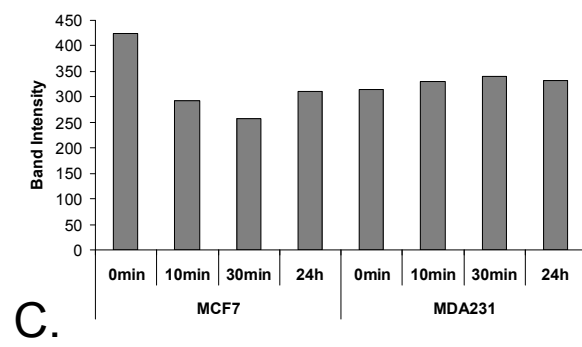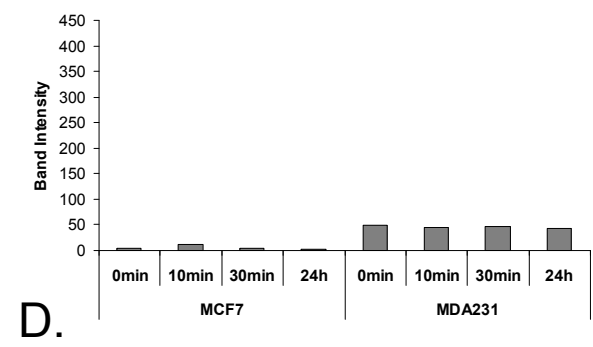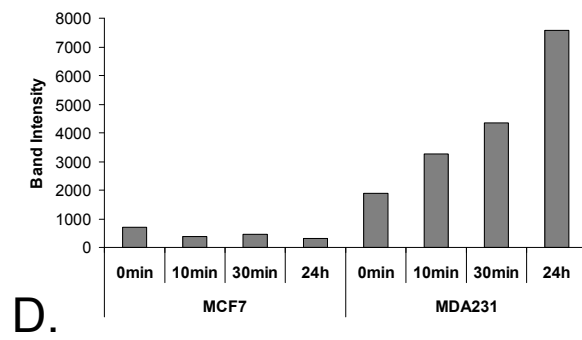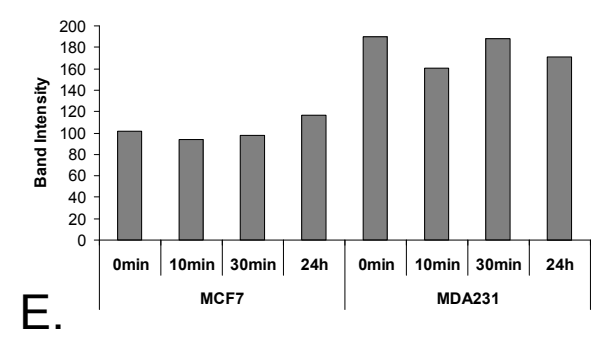

Supplement: Figure S5 — Quantification of Western blot analysis. Basal levels of Met are 1.56 times higher in MDA231 as compared to MCF7 cells and treatment with HGF/SF did not significantly change Met basal levels in either both cell lines (A). Levels of pMet are 6.4 times higher in MDA231 as compared to MCF7 cells. Sixty minutes following treatment with HGF/SF, pMet levels are 1.86 higher in MCF7 as compared to base line, but are still significantly lower than in MDA231, whose pMet levels did not significantly change following treatment with HGF/SF (B). Levels of ERK in MCF7 and MDA231 are similar (C). Levels of pERK are 15 times higher in MDA231 as compared to MCF7 cells. Levels of pERK did not significantly change following treatment with HGF/SF (D). As expected, E-cadherin levels are almost undetected in MCF7 cells and in MDA231, its levels are elevated following treatment with HGF/SF (E). Survivin levels are 1.9 higher in MDA231 as compared to MCF7 cells and do not significantly change following treatment with HGF/SF (F). (PDF) [file pone.0045969.s005.pdf]

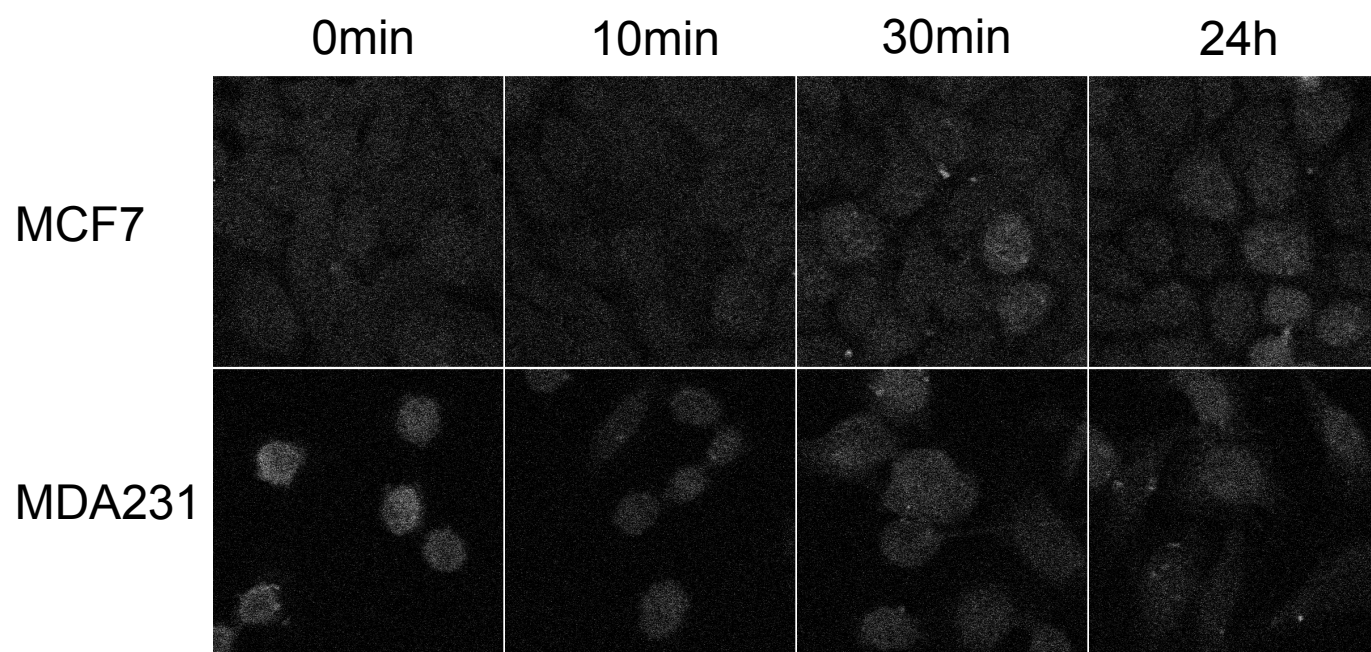

Supplement: Figure S6 — Survivin immuno-fluorescence in MCF7 and MDA231 cells. MCF7 and MDA231 cells were incubated with the primary antibody anti-Survivin (Santa Cruz, 1∶50). Slides were analyzed using a 510 Meta Zeiss confocal laser scanning microscope (CLSM). When comparing fluorescence intensities, identical CLSM parameters (e.g. pin hole, scanning line, laser light, contrast and brightness) were used. To compare the relative levels of protein expression, we used the average area intensity (AAI) image analysis procedure for cells immunostaining. The image analysis calculations were performed on five to ten microscopic fields. Cell outline was drawn based on DIC images; nuclei were defined based on the DAPI staining. Average pixel intensity was calculated separately for the nucleus and cytoplasm areas. (MICA software; Cytoview, Petach Tikva, Israel). Variance was analyzed by student’s T-test. (PDF) [file pone.0045969.s006.pdf]

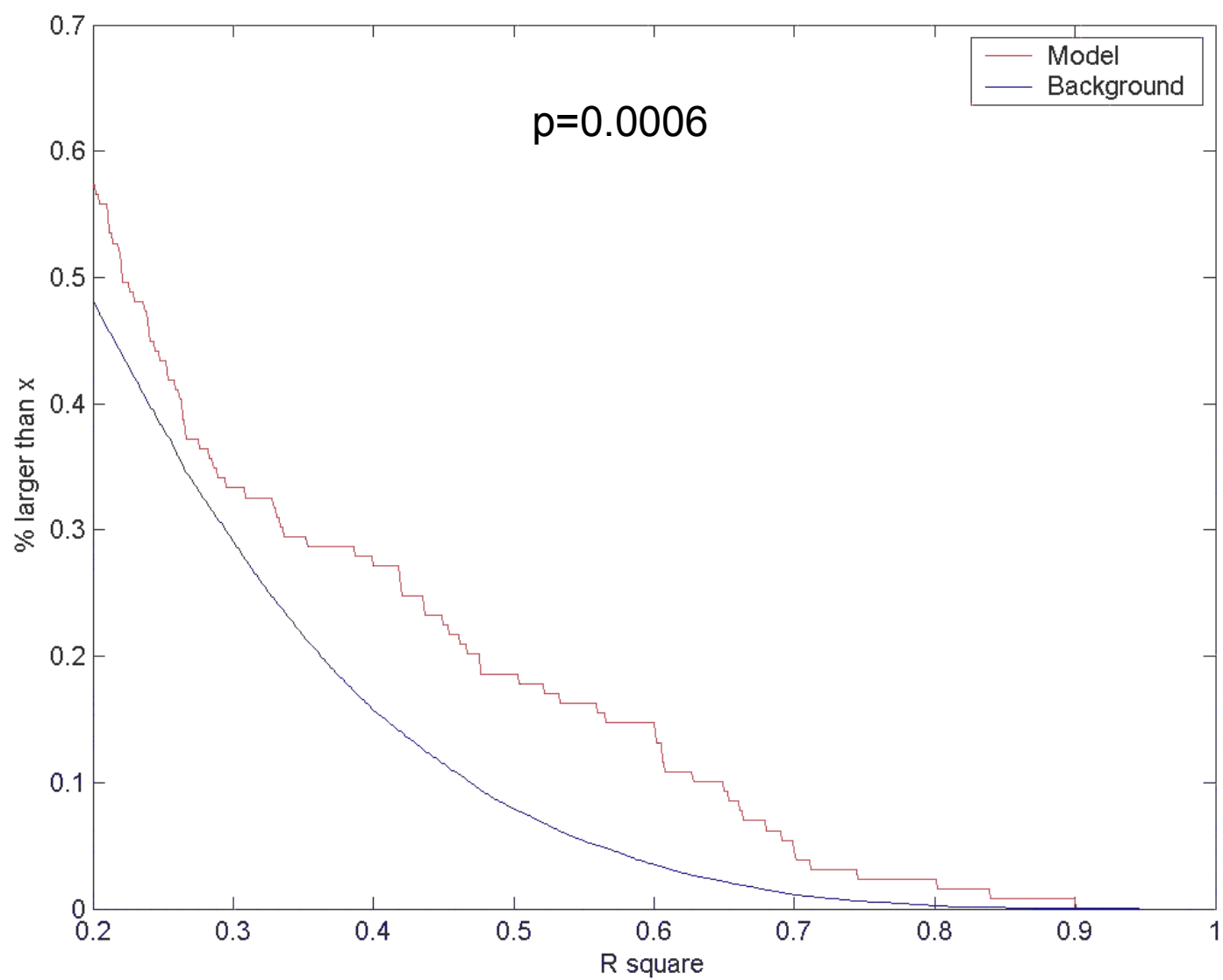

Supplement: Figure S7 — Gene-pair correlation distribution comparison of ANAT derived Met network. The gene-pair correlation distribution comparison of ANAT derived Met network is significantly higher then that of all the interacting genes in the ANAT database (p = 0.0006). (PDF) [file pone.0045969.s007.pdf]

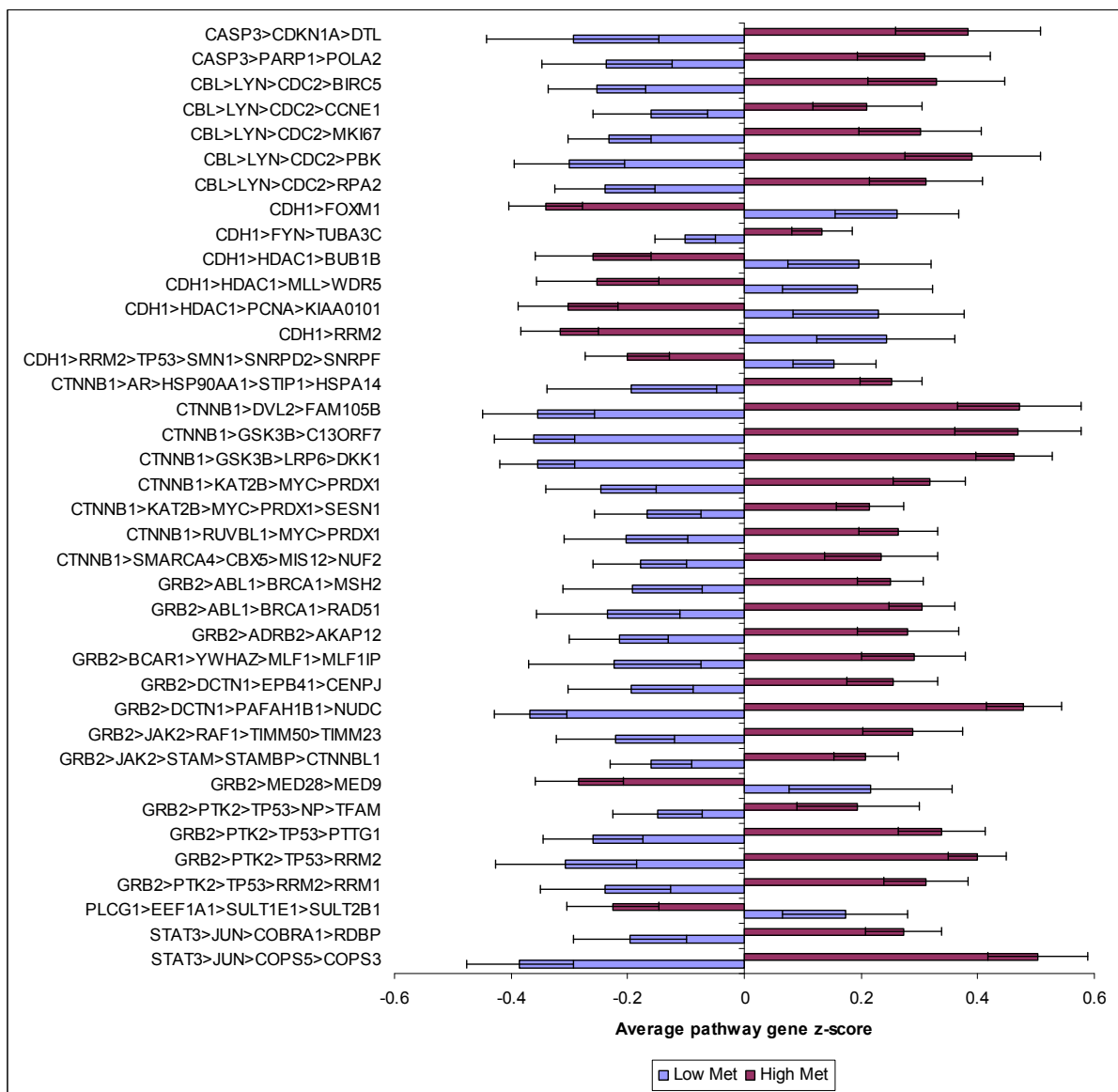

Supplement: Figure S8 — Expression score of ANAT-derived pathways. The expression score of 38 out of the 104 calculated pathways (36.5%) significantly differentiated High vs. Low Met cell lines as compared to a random expectation of 5% (p<1e-4, 30 highly expressed and 8 low/poorly expressed). (PDF) [file pone.0045969.s008.pdf]

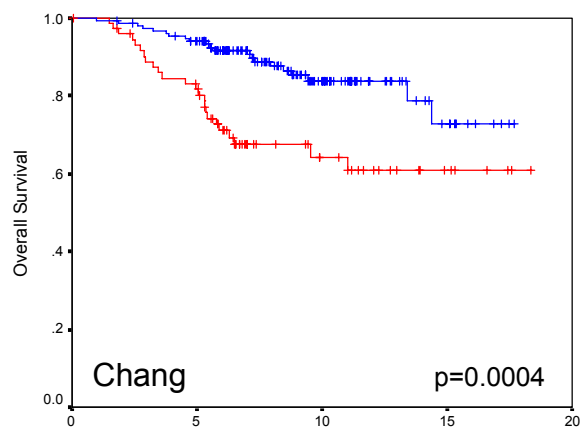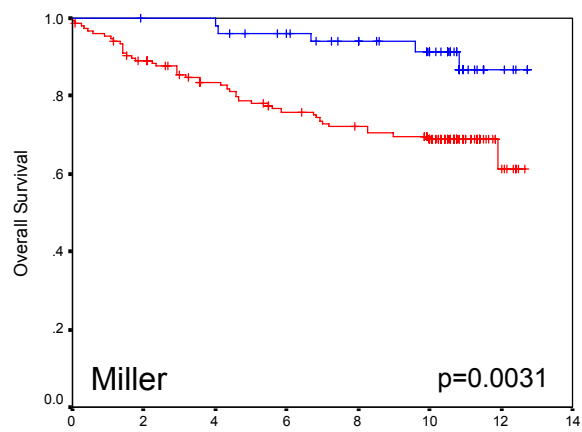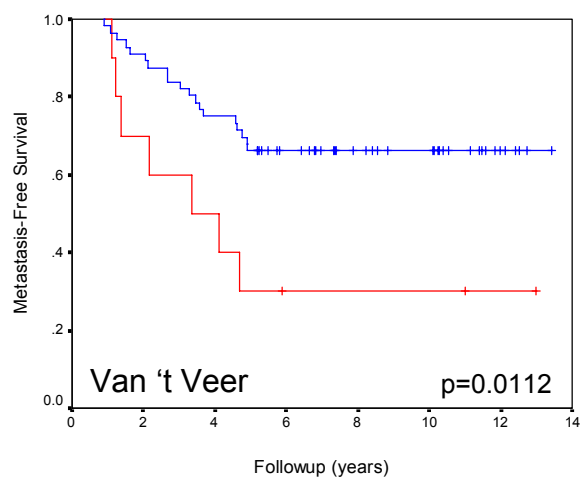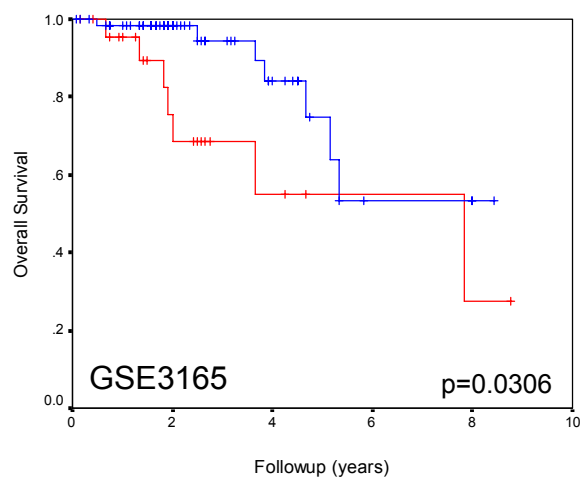

Supplement: Figure S9 — Subgroup analysis of Met kinetic signature by ER status. Subgroup analysis of Met kinetic signature by ER status in van ‘t Veer, Miller, Chang and GSE3165 data sets showed that high-Met kinetic signature correlated with poor prognosis in ER+ patients, but not in ER- patients. (PDF) [file pone.0045969.s009.pdf]
