# Supplementary material for: Met Kinetic Signature Derived from the Response to HGF/SF in a Cellular Model Predicts Breast Cancer Patient Survival
Source: PLoS One. 2012 Sep 25;7(9):e45969. doi: 10.1371/journal.pone.0045969 (PMC3457970; doi:10.1371/journal.pone.0045969)
Supplement: Table S1 — Met Kinetic Signature genes. (PDF) [file pone.0045969.s010.pdf]

Entrez ID	Symbol	Gane Name
332	SURVIVIN	baculoviral IAP repeat-containing 5
516	ATP5G1	ATP synthase, H+ transporting, mitochondrial F0 complex, subunit C1 (subunit 9)
699	BUB1	budding uninhibited by benzimidazoles 1 homolog (yeast)
701	BUB1B	budding uninhibited by benzimidazoles 1 homolog beta (yeast)
890	CCNA2	cyclin A2
898	CCNE1	cyclin E1
1062	CENPE	centromere protein E, 312kDa
1633	DCK	deoxycytidine kinase
1763	DNA2L	DNA replication helicase 2 homolog (yeast)
1870	E2F2	E2F transcription factor 2
1964	EIF1AX	eukaryotic translation initiation factor 1A, X-linked
2030	SLC29A1	solute carrier family 29 (nucleoside transporters), member 1
2237	FEN1	flap structure-specific endonuclease 1
2305	FOXM1	forkhead box M1
3014	H2AFX	H2A histone family, member X
3070	HELLS	helicase, lymphoid-specific
3112	HLA-DOB	major histocompatibility complex, class II, DO beta
3312	HSPA8	heat shock 70kDa protein 8
3336	HSPE1	heat shock 10kDa protein 1 (chaperonin 10)
3832	KIF11	kinesin family member 11
3838	KPNA2	karyopherin alpha 2 (RAG cohort 1, importin alpha 1); karyopherin alpha-2 subunit like
4085	MAD2L1	MAD2 mitotic arrest deficient-like 1 (yeast)
4131	MAP1B	microtubule-associated protein 1B
4172	MCM3	minichromosome maintenance complex component 3
4288	MKI67	antigen identified by monoclonal antibody Ki-67
4436	MSH2	mutS homolog 2, colon cancer, nonpolyposis type 1 (E. coli)
4830	NME1	non-metastatic cells 1, protein (NM23A) expressed in; NME1-NME2 readthrough transcript; non-metastatic cells 2, protein (NM23B) expressed in
5036	PA2G4	proliferation-associated 2G4, 38kDa; proliferation-associated 2G4 pseudogene 4
5052	PRDX1	peroxiredoxin 1
5422	POLA1	polymerase (DNA directed), alpha 1, catalytic subunit
5427	POLE2	polymerase (DNA directed), epsilon 2 (p59 subunit)
5481	PPID	peptidylprolyl isomerase D
5557	PRIM1	primase, DNA, polypeptide 1 (49kDa)
5888	RAD51	RAD51 homolog (RecA homolog, E. coli) (S. cerevisiae)
5984	RFC4	replication factor C (activator 1) 4, 37kDa
6118	RPA2	replication protein A2, 32kDa
6240	RRM1	ribonucleotide reductase M1
6241	RRM2	ribonucleotide reductase M2 polypeptide
6627	SNRPA1	small nuclear ribonucleoprotein polypeptide A'
6636	SNRPF	small nuclear ribonucleoprotein polypeptide F
6820	SULT2B1	sulfotransferase family, cytosolic, 2B, member 1
7019	TFAM	transcription factor A, mitochondrial
7278	TUBA3C	tubulin, alpha 3d; tubulin, alpha 3c
7372	UMPS	uridine monophosphate synthetase
7936	RDBP	RD RNA binding protein
8317	CDC7	cell division cycle 7 homolog (S. cerevisiae)
8318	CDC45L	CDC45 cell division cycle 45-like (S. cerevisiae)
8533	COPS3	COP9 constitutive photomorphogenic homolog subunit 3 (Arabidopsis)
8884	SLC5A6	solute carrier family 5 (sodium-dependent vitamin transporter), member 6
9055	PRC1	protein regulator of cytokinesis 1
9128	PRPF4	PRP4 pre-mRNA processing factor 4 homolog (yeast)
9232	PTTG1	pituitary tumor-transforming 1; pituitary tumor-transforming 2

9319	TRIP13	thyroid hormone receptor interactor 13
9374	PPT2	palmitoyl-protein thioesterase 2
9538	EI24	etoposide induced 2.4 mRNA
9590	AKAP12	A kinase (PRKA) anchor protein 12
9768	KIAA0101	KIAA0101
9787	DLGAP5	discs, large (Drosophila) homolog-associated protein 5
9816	URB2	URB2 ribosome biogenesis 2 homolog (S. cerevisiae)
9908	G3BP2	GTPase activating protein (SH3 domain) binding protein 2
10419	PRMT5	protein arginine methyltransferase 5
10426	TUBGCP3	tubulin, gamma complex associated protein 3
10431	TIMM23	translocase of inner mitochondrial membrane 23 homolog (yeast); translocase of inner mitochondrial membrane 23 homolog B (yeast)
10436	EMG1	EMG1 nucleolar protein homolog (S. cerevisiae)
10452	TOMM40	translocase of outer mitochondrial membrane 40 homolog (yeast)
10528	NOP56	NOP56 ribonucleoprotein homolog (yeast)
10606	PAICS	phosphoribosylaminoimidazole carboxylase, phosphoribosylaminoimidazole succinocarboxamide synthetase
10694	CCT8	similar to chaperonin containing TCP1, subunit 8 (theta); chaperonin containing TCP1, subunit 8 (theta)
10726	NUDC	nuclear distribution gene C homolog (A. nidulans)
10969	EBNA1BP2	EBNA1 binding protein 2
11091	WDR5	WD repeat domain 5
11130	ZWINT	ZW10 interactor
22918	CD93	CD93 molecule
22943	DKK1	dickkopf homolog 1 (Xenopus laevis)
23178	PASK	PAS domain containing serine/threonine kinase
23310	NCAPD3	
23397	NCAPH	non-SMC condensin I complex, subunit H
23649	POLA2	polymerase (DNA directed), alpha 2 (70kD subunit)
26589	MRPL46	mitochondrial ribosomal protein L46
27101	CACYBP	similar to calcyclin binding protein; calcyclin binding protein
27244	SESN1	sestrin 1
27258	LSM3	LSM3 homolog, U6 small nuclear RNA associated (S. cerevisiae); similar to Lsm3 protein
28960	DCPS	decapping enzyme, scavenger
28965	SLC27A6	solute carrier family 27 (fatty acid transporter), member 6
29089	UBE2T	ubiquitin-conjugating enzyme E2T (putative)
29090	C18ORF55	chromosome 18 open reading frame 55
29901	SAC3D1	SAC3 domain containing 1
51010	EXOSC3	exosome component 3
51053	GMNN	geminin, DNA replication inhibitor
51182	HSPA14	heat shock 70kDa protein 14
51514	DTL	denticleless homolog (Drosophila)
51728	POLR3K	polymerase (RNA) III (DNA directed) polypeptide K, 12.3 kDa
54148	MRPL39	mitochondrial ribosomal protein L39
54478	FAM64A	family with sequence similarity 64, member A
55038	CDCA4	cell division cycle associated 4
55055	ZWILCH	Zwilch, kinetochore associated, homolog (Drosophila)
55071	C9ORF40	chromosome 9 open reading frame 40
55090	MED9	mediator complex subunit 9
55276	PGM2	phosphoglucomutase 2
55789	DEPDC1B	DEP domain containing 1B
55835	CENPJ	centromere protein J
55872	PBK	PDZ binding kinase
56259	CTNNBL1	catenin, beta like 1

57104	PNPLA2	patatin-like phospholipase domain containing 2
64785	FLJ13912	GINS complex subunit 3 (Psf3 homolog)
65008	MRPL1	mitochondrial ribosomal protein L1
79019	CENPM	centromere protein M
79081	MGC2477	
79084	WDR77	WD repeat domain 77
79596	C13ORF7	ring finger protein 219
79682	MLF1IP	MLF1 interacting protein
79723	SUV39H2	suppressor of variegation 3-9 homolog 2 (Drosophila)
79733	E2F8	E2F transcription factor 8
80119	C15ORF20	PIF1 5'-to-3' DNA helicase homolog (S. cerevisiae)
80179	MYOHD1	myosin XIX
81610	FAM83D	family with sequence similarity 83, member D
81892	C14ORF156	chromosome 14 open reading frame 156
83540	NUF2	NUF2, NDC80 kinetochore complex component, homolog (S. cerevisiae)
84315	MON1A	MON1 homolog A (yeast)
84811	BUD13	BUD13 homolog (S. cerevisiae)
84908	FAM136A	family with sequence similarity 136, member A
90135	BTBD6	BTB (POZ) domain containing 6
90268	FAM105B	family with sequence similarity 105, member B
93323	NY-SAR-48	HAUS augmin-like complex, subunit 8
113457	TUBA3D	tubulin, alpha 3d; tubulin, alpha 3c
152579	SCFD2	sec1 family domain containing 2
157313	CDCA2	cell division cycle associated 2
200895	DHFRL1	dihydrofolate reductase-like 1
317786	317786	
378708	APITD1	cortistatin; apoptosis-inducing, TAF9-like domain 1
642981	642981	
