# Supplementary material for: Met Kinetic Signature Derived from the Response to HGF/SF in a Cellular Model Predicts Breast Cancer Patient Survival
Source: PLoS One. 2012 Sep 25;7(9):e45969. doi: 10.1371/journal.pone.0045969 (PMC3457970; doi:10.1371/journal.pone.0045969)
Supplement: Table S2 — Primers for qRT-PCR. The following primers are used for the quantification of gene expression. (PDF) [file pone.0045969.s011.pdf]

	Forward & Reverse Primers:
GAPDH	TGCACCACCAACTGCTTAGC<br>GGCATGGACTGTGGTCATGAG
Met	CAGAGACTTGGCTGCAAGAA<br>GGCAAGACCAAAATCAGCA
Survivin	GAACTGGCCCTTCTTGGAG<br>AAGTCTGGCTCGTTCTCAGTG
Cyclin E1	ATCAGCACTTTCTTGAGCAACA<br>TTGTGCCAAGTAAAAGGTCTCC
Ki67	AGACGCCTGGTTACTATCAAAAG<br>GGAAGCTGGATACGGATGTCA
Pbk	AGGAGTCTCTCTACCACTGGA<br>GGCAAATATGTCTGCCTTGTCAG
