# Supplementary material for: Met Kinetic Signature Derived from the Response to HGF/SF in a Cellular Model Predicts Breast Cancer Patient Survival
Source: PLoS One. 2012 Sep 25;7(9):e45969. doi: 10.1371/journal.pone.0045969 (PMC3457970; doi:10.1371/journal.pone.0045969)
Supplement: Table S3 — ANAT derived pathways originating from Met to the kinetic signature genes. (PDF) [file pone.0045969.s012.pdf]

Anat Pathway

MET,CASP3,CDKN1A,DTL
MET,CASP3,HSPE1
MET,CASP3,PARP1,POLA2
MET,CBL,LYN,CDC2,SURVIVIN
MET,CBL,LYN,CDC2,CCNE1
MET,CBL,LYN,CDC2,KIF11
MET,CBL,LYN,CDC2,MKI67
MET,CBL,LYN,CDC2,PBK
MET,CBL,LYN,CDC2,RPA2
MET,CBL,PTK2B,EWSR1,SUV39H2
MET,CBL,UBE2L3,UBE3A,MCM7,CDC45L
MET,CBL,UBE2L3,UBE3A,MCM7,MCM2,CDC7
MET,CBL,UBE2L3,UBE3A,MCM7,MCM3
MET,CDH1,ACTG1,PRSS23,NCAPD3
MET,CDH1,FOXM1
MET,CDH1,FYN,TUBA3C
MET,CDH1,HDAC1,BUB1
MET,CDH1,HDAC1,BUB1B
MET,CDH1,HDAC1,BUB1B,CENPE
MET,CDH1,HDAC1,BUB1B,MAD2L1
MET,CDH1,HDAC1,DDX17,EXOSC3
MET,CDH1,HDAC1,MLL,WDR5
MET,CDH1,HDAC1,PCNA,CDT1,GMNN
MET,CDH1,HDAC1,PCNA,FEN1
MET,CDH1,HDAC1,PCNA,KIAA0101
MET,CDH1,HDAC1,PCNA,RFC4
MET,CDH1,HDAC1,PCNA,XRCC5,PRIM1
MET,CDH1,HDAC2,CHD3,SLC27A6
MET,CDH1,HDAC2,SP3,STK19,SLC29A1
MET,CDH1,RRM2
MET,CDH1,RRM2,RRM1
MET,CDH1,RRM2,TP53,NP,TFAM
MET,CDH1,RRM2,TP53,PTTG1
MET,CDH1,RRM2,TP53,SMN1,SNRPD2,CDC5L,SNRPA1
MET,CDH1,RRM2,TP53,SMN1,SNRPD2,LSM2,LSM3
MET,CDH1,RRM2,TP53,SMN1,SNRPD2,SNRPF
MET,CTNNB1,AR,CALM1,AKAP9,TUBGCP3
MET,CTNNB1,AR,HSP90AA1,STIP1,HSPA14
MET,CTNNB1,AR,SART3,PRPF4
MET,CTNNB1,AR,UBE2I,IPO13,EIF1AX
MET,CTNNB1,BTRC,DLGAP5
MET,CTNNB1,CACYBP
MET,CTNNB1,DVL2,FAM105B
MET,CTNNB1,GSK3B,C13ORF7
MET,CTNNB1,GSK3B,LRP6,DKK1
MET,CTNNB1,IKBKB,AURKA,NME1
MET,CTNNB1,IKBKB,NFKBIA,G3BP2
MET,CTNNB1,KAT2B,MYC,PRDX1
MET,CTNNB1,KAT2B,MYC,PRDX1,SESN1
MET,CTNNB1,RUVBL1,MYC,PRDX1

MET,CTNNB1,RUVBL1,MYC,PRDX1,SESN1
MET,CTNNB1,SMAD7,BMPR1A,BMPR2,NOP56
MET,CTNNB1,SMARCA4,CBX5,MIS12,NUF2
MET,CTNNB1,SMARCA4,CBX5,MIS12,ZWINT
MET,GRB2,ABL1,BRCA1,CCNA2
MET,GRB2,ABL1,BRCA1,GFI1B,MON1A
MET,GRB2,ABL1,BRCA1,GFI1B,RINT1,ZW10,ZWILCH
MET,GRB2,ABL1,BRCA1,H2AFX
MET,GRB2,ABL1,BRCA1,MSH2
MET,GRB2,ABL1,BRCA1,RAD50,RINT1,GFI1B,MON1A
MET,GRB2,ABL1,BRCA1,RAD50,RINT1,ZW10,ZWILCH
MET,GRB2,ABL1,BRCA1,RAD51
MET,GRB2,ABL1,RB1,E2F2
MET,GRB2,ABL1,RB1,PA2G4
MET,GRB2,ABL1,RB1,POLA1
MET,GRB2,ADRB2,AKAP12
MET,GRB2,BCAR1,YWHAZ,MLF1,MLF1IP
MET,GRB2,CDKN1B,CDK2,PPP2CA,CCT8
MET,GRB2,CDKN1B,CDK2,PRC1
MET,GRB2,DCTN1,EPB41,CENPJ
MET,GRB2,DCTN1,EPB41,PAICS
MET,GRB2,DCTN1,PAFAH1B1,MAP1B
MET,GRB2,DCTN1,PAFAH1B1,NUDC
MET,GRB2,ITK,KPNA2
MET,GRB2,JAK2,PRMT5
MET,GRB2,JAK2,PRMT5,WDR77
MET,GRB2,JAK2,RAF1,TIMM50,TIMM23
MET,GRB2,JAK2,STAM,STAMBP,CTNNBL1
MET,GRB2,KHDRBS1,EMG1
MET,GRB2,KIT,CD63,HLA-DOB
MET,GRB2,MED28,MED19,DCK
MET,GRB2,MED28,MED9
MET,GRB2,PRKAR1A,ARFGEF1,FBL,PASK
MET,GRB2,PTK2,TP53,NP,TFAM
MET,GRB2,PTK2,TP53,PTTG1
MET,GRB2,PTK2,TP53,RRM2
MET,GRB2,PTK2,TP53,RRM2,RRM1
MET,GRB2,PTK2,TP53,SMN1,SNRPD2,CDC5L,SNRPA1
MET,GRB2,PTK2,TP53,SMN1,SNRPD2,LSM2,LSM3
MET,GRB2,PTK2,TP53,SMN1,SNRPD2,SNRPF
MET,GRB2,USP8,OTUB1,EBNA1BP2
MET,GRB2,WAS,CDC42,CDC42EP3,UMPS
MET,ITGB4,PRKCA,LMNA,URB2
MET,PLCG1,BAG3,HSPA8
MET,PLCG1,BAG3,HSPA8,POLR3D,POLR3K
MET,PLCG1,BAG3,HSPA8,PPID
MET,PLCG1,EEF1A1,MAD2L1BP,TRIP13
MET,PLCG1,EEF1A1,POLE2
MET,PLCG1,EEF1A1,SULT1E1,SULT2B1
MET,RANBP10,RAN,RANBP1,CD93
MET,RANBP9,SMAD9,PNPLA2

MET,RANBP9,USP11,CIAO1,HELLS
MET,STAT3,JUN,COBRA1,RDBP
MET,STAT3,JUN,COPS5,COPS3
