# Supplementary material for: Met Kinetic Signature Derived from the Response to HGF/SF in a Cellular Model Predicts Breast Cancer Patient Survival
Source: PLoS One. 2012 Sep 25;7(9):e45969. doi: 10.1371/journal.pone.0045969 (PMC3457970; doi:10.1371/journal.pone.0045969)
Supplement: Table S4 — Differences in Met canonical pathway expression score between patient groups segmented by Met kinetic signature. (PDF) [file pone.0045969.s013.pdf]

Cohort	High Met canonical pathway activity	Low Met canonical pathway activity	p
Miller	0.013±0.02	-0.041±0.03	NS
van 't Veer	0.111±0.03	-0.084±0.03	<0.0001
Chang	0.085±0.02	-0.066±0.02	<0.0001
GSE3165	0.047±0.02	-0.047±0.03	0.005
GSE11121	0.027±0.02	-0.028±0.02	NS
GSE1456	0.036±0.03	-0.044±0.02	0.025
