# Supplementary material for: Met Kinetic Signature Derived from the Response to HGF/SF in a Cellular Model Predicts Breast Cancer Patient Survival
Source: PLoS One. 2012 Sep 25;7(9):e45969. doi: 10.1371/journal.pone.0045969 (PMC3457970; doi:10.1371/journal.pone.0045969)
Supplement: Table S5 — Cox proportional hazards regression survival analysis using Met kinetic signature and basal-like classification on three breast cancer patient cohorts. (PDF) [file pone.0045969.s014.pdf]

Cohort	Basal-type Classification Parameter	HR (CI 95%)	*p*	*p* overall	Histological Grade Parameter	HR (CI 95%)	*p*	*p* overall	Estrogen Receptor Status Parameter	HR (CI 95%)	*p*	*p* overall
Chang	Basal	1.89 (1.11, 3.21)	0.019	<0.0001	Grade		0.0002	<0.0001	ER	0.41 (0.25, 0.68)	0.0005	<0.0001
	Kinetic	2.24 (1.36, 3.68)	0.001		Grade(1)	0.13 (0.04, 0.36)	0.0001		Kinetic	1.93 (1.16, 3.22)	0.011	
					Grade(2)	0.55 (0.33, 0.92)	0.022					
					Kinetic	1.66 (1.01, 2.72)	0.046					
Miller					Grade		0.005	<0.0001	ER	1.28 (0.55, 2.99)	NS	0.008
					Grade(1)	2.48 (0.32, 19.13)	NS		Kinetic	3.84 (1.53, 9.66)	0.004	
					Grade(2)	0.26 (0.11, 0.58)	0.001					
					Grade(3)	0.53 (0.29, 0.96)	0.036					
					Kinetic	3.66 (1.44, 9.33)	0.007					
van 't Veer					Grade		NS	0.001	ER	0.61 (0.3, 1.27)	NS	0.003
					Grade(1)	0 (0, 3.4E+225)	NS		Kinetic	1.85 (0.9, 3.83)	NS	
					Grade(2)	0.46 (0.22, 0.96)	0.039					
					Kinetic	Not added to model						
GSE11121					Grade		NS	0.036				
					Grade(1)	0.33 (0.1, 1.09)	NS					
					Grade(2)	0.56 (0.28, 1.09)	NS					
					Kinetic	1.43 (0.75, 2.71)	NS					
GSE3165	Basal	1.12 (0.54, 2.33)	NS	0.013	Grade		NS	0.016	ER	0.53 (0.26, 1.08)	NS	0.014
	Kinetic	2.39 (1.15, 4.96)	0.019		Grade(1)	0.6 (0.21, 1.76)	NS		Kinetic	1.92 (0.95, 3.88)	NS	
					Grade(2)	0.19 (0.03, 1.42)	NS					
					Grade(3)	0.79 (0.39, 1.61)	NS					
					Kinetic	2.46 (1.24, 4.87)	0.01					
GSE1456	Basal	1.14 (0.54, 2.43)	NS	<0.0001	Grade	0 (0, 0)	NS	0.001				
	Kinetic	4.28 (1.87, 9.8)	0.001		Grade(1)	0.53 (0.11, 2.46)	NS					
					Grade(2)	1.24 (0.61, 2.54)	NS					
					Kinetic	0.2 (0.07, 0.55)	0.002					
