# Supplementary material for: Met Kinetic Signature Derived from the Response to HGF/SF in a Cellular Model Predicts Breast Cancer Patient Survival
Source: PLoS One. 2012 Sep 25;7(9):e45969. doi: 10.1371/journal.pone.0045969 (PMC3457970; doi:10.1371/journal.pone.0045969)
Supplement: Text S1 — Supplemental Methods. (DOC) [file pone.0045969.s015.doc]

## Supplementary Methods

### If not otherwise specified reagents were purchased from Sigma, Rehovot, Israel

**Characterizing high vs. low Met expressing cell lines**

**Western-blot analysis** Cells were washed twice with cold PBS and lysed in 0.5 ml lysis buffer (20 mM Tris-HCl, pH 7.8, 100 mM NaCl, 50 mM NaF, 1% NP40, 0.1% SDS, 2 mM EDTA, 10% glycerol) with protease inhibitor cocktail (Boehringer Mannheim, Germany) and 1 mM sodium orthoVanadate. The samples were subjected to western blot (WB) analysis using antibodies against: ERK K-23 (Santa Cruz Biotechnology, Inc. Santa Cruz, CA, USA, 1:500), p-ERK E-4 (Santa-Cruz, 1:500), E-Cadherin (Cell Signaling Technology, Inc. Danvers, MA, USA, 1:1,000), Survivin (Santa Cruz 1:200) or Actin C4 (Millipore, 1:5,000). Visualization was achieved using Peroxidase-conjugated anti-mouse IgG antibodies (Jackson ImmunoResearch Laboratories, West Grove, PA, USA, 1:20,000) or Peroxidase-conjugated anti-rabbit IgG antibodies (Jackson ImmunoResearch Laboratories, 1:20,000), ECL reaction and exposure to X-ray Film was according to manifacture instractions (Fuji, Tokyo, Japan). Other blots were visualized and quantification performed using the Odyssey Infrared Imaging System (LI-COR, Inc., Lincoln, NE, USA). For the Odyssey, membranes were incubated with fluorescent-labeled secondary antibody at 1:10,000 dilution with 3% BSA in PBS for 30-min protected from light. After washing in PBS ‏0.1% Tween-20, the membranes were scanned using an Odyssey Infrared Imaging System.

### Immunofluorescence Analysis

The cells were washed twice with PBS and fixed for 10 minutes at room temperature in 100% methanol permeabilized for 10 minutes in 100% acetone and washed in PBS. Blocking was done in 10% normal donkey serum/ PBS for 10 minutes at room temperature. The cells were subsequently incubated with the primary antibody anti-Survivin (Santa Cruz, 1:50) and incubated over night at 4ºC. The cells were then washed three times in PBS and incubated for 1 hour in the dark at room temperature with the secondary antibody (FITC-conjugated donkey anti-rabbit IgG diluted 1:150 in PBS, Jackson ImmunoResearch Laboratories). Slides were then washed in PBS and mounted with cover-slips using Fluorescent Mounting Medium (Golden Bridge International inc., WA, USA).

The samples were analyzed using a 510 Meta Zeiss confocal laser scanning microscope (CLSM) with the following configuration: 25 mW Krypton/Argon (488nm) and HeNe (633nm) laser lines. When comparing fluorescence intensities, identical CLSM parameters (e.g. pin hole, scanning line, laser light, contrast and brightness) were used. To compare the relative levels of protein expression, we used the average area intensity (AAI) image analysis procedure for cells immunostaining. The image analysis calculations were performed on five to ten microscopic fields. Cell outline was drawn based on DIC images; nuclei were defined based on the DAPI staining. Average pixel intensity was calculated separately for the nucleus and cytoplasm areas. (MICA software; Cytoview, Petach Tikva, Israel). Variance was analyzed by student's T-test.

### cDNA microarrays hybridization using HGF/SF treated human breast cancer cell lines

The six cell lines were starved for 24h (0.1% FBS) and treated with 100ng/ml HGF/SF for 0, 10 min, 30 min, and 24 hours; Total RNA was isolated from cells using TRI reagent (Sigma), according to the manufacturer's instructions. The quality and quantities of RNA samples were verified using agarose gel electrophoresis.

**cDNA microarrays**. cDNA clones of the Sequence Validated Human cDNA Library (ResGen, Invitrogen) were amplified by PCR using primers complementary to the vector sequences. The purified PCR products were then robotically arrayed onto polylysine-coated microarray slides, on which the DNAs were immobilized by UV light. Of the cDNA microarrays described here, 19,968 contained sequence-validated human cDNAs, generally with insert sizes of 0.25–2.5 kb. Among them, 111 cDNA clones were printed twice on each slide (VAI microarray facilities, Grand Rapids, MI, USA).

**Obtaining relative expression levels**. Untreated normal human breast cell line (MCF10) served as reference for the 6 human breast cancer cell lines. All cell lines RNA was labeled twice, once with Cy3 and once with Cy5. For each treated cell line RNA labeled using Cy3, the reference was labeled with Cy5, and for each treated cell line RNA labeled using Cy5, the reference was labeled with Cy3. These reciprocal experiments were done to control any differences caused by the labeling dye. After purification through a 30 Microcon-Amicon filter (Millipore Corporation. Billerica, MA, USA), the Cy3- and Cy5-labeled cDNA probes were mixed with 40 μg human Cot-1 DNA (Gibco, Tulsa OK, USA), 20 μg yeast tRNA (GIBCO) and 20 μg poly d(A). The mixture was concentrated to 16 μl with a Microcon-30 filter (Amicon). After denaturation, 16 μl of 2× hybridization solution (50% formamide, 10× SSC, 0.2% SDS) was added into the mixture and incubated for at least 20 min at 42°C. The mixture was then hybridized onto the pre-warmed (42°C) microarray slides for 18 h at 42°C.

Arrays were scanned in a fluorescence confocal scanner (Scan Array Lite, GSI Lumonics, Billerica, CA). Images were analyzed using GenePix Pro 3.0 software (Axon Instruments, Burlingame, CA). Spots were defined by the automatic grid feature of the software and adjusted manually when necessary.

For each cDNA spot, the local background was subtracted from the total signal intensities of Cy5 and Cy3. The ratio of net fluorescence from the Cy5-specific channel to that from the Cy3-specific channel was calculated for each spot. This ratio represents the expression of the cDNA in the cells treated with HGF/SF relative to the expression in normal breast cells (MCF10) without treatment. Six independent experiments were performed to reduce variations related to labeling and hybridization efficiencies among the experiments.

### Data preparation

Gene values in samples which were labeled with Cy3 and the reference in Cy5 – were converted to the minus of their value. Gene IDs were converted to Entrez ID and genes without corresponding Entrez IDs were removed from analysis. In case of more than one gene entry per Entrez ID, the one with the highest standard deviation was selected and the rest was removed from analysis.

Samples from the cell line SkLMS were not used for analysis in this work because of inconsistent data regarding Met activity in this cell line.

### Quantitative real-time PCR

Total cellular RNA was isolated from cell cultures using TRI reagent. cDNA was synthesized using High Capacity cDNA Reverse Transcription kits. mRNA expression of certain genes was evaluated by ABI 9600 HT quantitative real time PCR system and compared to the housekeeping GAPDH gene. Quantification was done using the SYBR green PCR mix. All reagents were obtained from Applied Biosystems, Foster City, CA, USA. The primers used for the quantification of gene expression are listed in Table S2. The selected genes were independently determined using real-time PCR, in representative, low (MCF7) and high (MDA231) Met cell lines. Real-time PCR data were normalized to GAPDH. We chose GAPDH as the normalizing control because its expression remained unchanged between the different cell lines and treatments.

### Generating Met kinetic signature

To identify genes whose mRNA levels significantly changed at different time points following HGF/SF treatment, gene values were normalized with an average of 0 and a standard deviation of 1, per cell line. A two-tailed t-test was then performed for each gene between 0 min and the different time points (10 min, 30 min and 24 hours). The p-values were corrected for multiple testing using the False Discovery Rate (FDR) procedure [1] and results under a cutoff 1e-4 were considered significant. Genes with significant alteration were grouped according to the kinetics of alteration from baseline (0 min) into six signatures: "10-up" (“10-down”) – genes which were significantly up- (down-) regulated 10min after HGF/SF; "30-up" (“30-down”) – genes which responded after 30min and not included in the 10-up (10-down) group; and similarly - "24-up" (“24-down”) – for genes that responded after 24h. Each of the six groups was assessed for its ability to differentiate between high and low Met cell lines. To this end, we employed an unsupervised approach, by clustering the cell-lines into two groups based on the expression levels of the genes in each signature. Clustering was performed using the standard hierarchical method with complete linkage.

### Assessing mRNA half-life

We used the mRNA half life data base published by Sharova et al. [2] to assess the mRNA half life of Met canonical and Met kinetic signature genes. Genes who had a half life of zero were excluded from the analysis.

### Met canonical pathway genes [3]

MET, AKT1, AP1B1, CBL, CDC42, CRK, CRKL, DOCK1, ETS1, FAS, GAB1, GRB2, HRAS, INPP5D, KRAS, MAPK1, MAPK3, NRAS, PAK1, PIK3R1, PLCG1, PTPN11, RAC1, RAF1, RAP1A, RAP1B, RAPGEF1, SOS1, SOS2, SPRY2, SRC, STAT3

### Met inhibition - cellular model

To evaluate the association of the signature to Met inhibition, we used genome-wide expression measurements from the cellular model described by Bertotti et al. [4]. In brief, the cellular model included: GTL16, a gastric carcinoma cell line that contains 11 extra copies of the MET locus and EGFR-addicted (expressing a basally hyperactive EGFR ) colon carcinoma cells (DiFi), which were treated with: 1) a Met specific inhibitor (PHA-665752); 2) an inducible Met siRNA; 3) EGFR inhibitor (gefitinib) or 4) DMSO.

### Met activation animal model - transgenic mouse model expressing oncogenic type Met receptor

To further validate the specificity of the signature to Met activation, we used genome-wide expression measurements of normal and tumor samples taken from a transgenic mouse model, expressing oncogenic type Met receptor (Met^mt^) that develops a high incidence of diverse mammary tumors with basal characteristics (GSE10450) [5].

## Bibliography

1. Benjamini Y, Drai D, Elmer G, Kafkafi N, Golani I (2001) Controlling the false discovery rate in behavior genetics research. Behav Brain Res 125: 279-284.

2. Sharova LV, Sharov AA, Nedorezov T, Piao Y, Shaik N, et al. (2009) Database for mRNA half-life of 19 977 genes obtained by DNA microarray analysis of pluripotent and differentiating mouse embryonic stem cells. DNA Res 16: 45-58.

3. Birchmeier C, Birchmeier W, Gherardi E, Vande Woude GF (2003) Met, metastasis, motility and more. Nat Rev Mol Cell Biol 4: 915-925.

4. Bertotti A, Burbridge MF, Gastaldi S, Galimi F, Torti D, et al. (2009) Only a subset of met-activated pathways are required to sustain oncogene addiction. Sci Signal 2: er11.

5. Ponzo MG, Lesurf R, Petkiewicz S, O'Malley FP, Pinnaduwage D, et al. (2009) Met induces mammary tumors with diverse histologies and is associated with poor outcome and human basal breast cancer. Proc Natl Acad Sci U S A 106: 12903-12908.
