# Supplementary material for: Met Kinetic Signature Derived from the Response to HGF/SF in a Cellular Model Predicts Breast Cancer Patient Survival
Source: PLoS One. 2012 Sep 25;7(9):e45969. doi: 10.1371/journal.pone.0045969 (PMC3457970; doi:10.1371/journal.pone.0045969)
Supplement: Text S2 — Supplemental Results. (DOC) [file pone.0045969.s016.doc]

## Supplementary Results

### Enriched GO annotations and KEGG pathways in Met kinetic signature genes (p<1e-4)

Enriched gene ontology (GO) terms for this set include: cell division, cell cycle checkpoint, regulation of mitosis, spindle organization and biosynthesis, ribosome and nucleoside biosynthesis, DNA-dependent DNA replication and DNA repair. Enriched KEGG pathways of Met kinetic signature genes include: DNA replication, Pyrimidine metabolism, Cell cycle and Purine metabolism.

### Validation and specificity of Met kinetic signature

To evaluate whether the prognostic properties of Met kinetic signature is attributed to the cell cycle related genes, we removed cell cycle genes (according to their GO annotation) from the Met kinetic signature, resulting in a 96 gene signature. The reduced signature significantly correlated with Met activation animal model and Met inhibition cellular model and predicted survival in five of six large breast cancer patient cohorts (Figure S8).

### Significant GO annotations of the ANAT derived pathways (p<1e-4)

HGF/SF receptor signaling pathway (23%), Androgen receptor signaling pathway (3.8%), biopolymer metabolic process (3.8%), cell cycle process (3.8%), DNA damage response, signal transduction resulting in induction of apoptosis (3.8%), Negative regulation of growth (2.9%) and Wnt receptor signaling pathway (2.9%).

### Validation of the ANAT-derived network

We further tested whether using ANAT with the Met canonical pathway instead of the Met-kinetic signature will yield better prognostic values. We used ANAT to calculate the PPI pathways linking Met (acting as an anchor point) to the canonical pathway genes. The resulting “canonical” network contained 21 pathways. The expression score of 10 (46%) of the pathways significantly differentiate the High from the Low Met cell lines (higher rate than in the kinetic signature model). However, importantly none of the pathways in the canonical correlated with patient survival in all three breast cancer data sets.

### Assessing the specificity of Met kinetic signature to Met

The association between Met activation and basal-like tumors[1-4], as well as with ER-negative tumors [5,6] and high histological grade [7] is well known. To evaluate whether Met kinetic signature has prognostic value independent of the basal-type classification, ER status and histological grade, we performed Cox proportional hazards regression, using the patient cohorts which included Claudin classification (Chang, GSE3165 and GSE1456), ER status (van 't Veer, Miller, Chang and GSE3165) and histological grade (van 't Veer, Miller, Chang, GSE3165, GSE1456 and GSE11121) as well as prognostic data. Met kinetic signature correlated with patient survival independent of Basal-like classification in all three patient cohorts, ER status in two of four patient cohorts and histological grade in four of five patient cohorts (Table S5). We further show that Met kinetic signature significantly identifies patients with bad prognosis among ER(+) patients (Figure S9). These results demonstrate Met kinetic signatures specificity to Met.

Receptor and cell specificity result from the existence of a unique combination set of cell-cycle and other proteins at each level of signal transduction cascades used in a limited set of shared intracellular signal transduction pathways [8]. Based on this hypothesis we constructed a Met signature based on HGF/SF kinetic response in Met positive and negative breast cancer cell lines. The kinetic signature contained 35 cell cycle genes that some are known to be prognostic genes in breast cancer. Met kinetic signature without these genes is still prognostic for patient survival, although less than the original signature (Text S2).

### Sub-group analysis of the kinetic signature

Sub-group analysis by ER+/- in three data sets (van't Veer, Miller and Chang) showed in all three data sets that high-Met kinetic signature correlated with poor prognosis in ER+ patients, but had no prognostic effect in ER- patients (Figure S9). This demonstrates that Met kinetic signature identifies ER+ patients with poor prognosis.

### Met pathway signature vs. 70 gene signature

### We compared the prediction of five year survival between Met pathway signature and the 70-gene signature [9] on the Chang data set. Met pathway signature had sensitivity of 61%, specificity of 78%, positive predictive value (PPV) of 83% and negative predictive value (NPV) of 52% as compared to 70-gene signature which had a sensitivity of 91%, specificity of 52%, PPV of 98% and NPV of 43%.

**GRB2>BCAR1>YWHAZ>MLF1>MLF1IP pathway:**

The genes in this pathway are involved in cell migration, invasion, recovery from spindle damage, and correlate with anti-estrogen resistance and p53 deactivation [10-16]. Moreover, high mRNA levels of YWHAZ was shown to be associated with chemotherapy resistance and recurrence of breast cancer [17]. YWHAZ interacts with BCAR1, in a phospho-serine dependent manner [18], other genes were found to be associated by yeast two hybrid experiments [19]. Met, Src and their downstream substrate BCAR1 were shown to play a major role in PC3 migration and invasion [11] indicating that Met can activate this pathway.

**CBL>LYN>CDC2>(Survivin, Cyclin-E1, Ki67, PBK) pathways:**

Four out of the ANAT-derived Met pathways originate from the Cbl, Lyn, Cdc2 pathway leading to 4 different nodes (Survivin, Cyclin-E1, Ki67 and PBK). Cbl and Lyn were shown to be associated with proliferation and DNA synthesis, and are involved in cell cycle arrest signaling [20-23]. Cdc2 association with Lyn was demonstrated by immunoprecipitation essay [24]. Cdc2, Survivin, Cyclin-E1, Ki67 and PBK have important roles in cell cycle regulation, apoptosis inhibition and functional loss of p53 [25-32]. Moreover, Cyclin-E1 is associated with rastuzumab resistance in HER2+ breast cancer patients [33]. Alteration in the expression of different combinations of these genes is associated with tumor aggressiveness and poor outcome in several malignancies including stage-I breast cancer [27,28,34-44]. Moreover, Cyclin-E1 overexpression is a specific marker of triple-negative and basal-like tumors [42]. The role that this pathway plays in Met signaling is not documented. However, HGF/SF was shown to induce strong tyrosine phosphorylation of Cbl in B cells and increases its association with Lyn [45,46]. Co-expression of HGF/SF and Met in breast and other cancers correlate with high Ki67 expression levels [47,48]. Our results showing down regulation of Survivin mRNA levels upon HGF/SF treatment are in agreement with reports that HGF/SF treatment is associated with reduced mRNA levels of Survivin in KG-1 and HepG-2 cell lines [49]. Moreover, we show that the HGF/SF induced kinetics in Survivin's mRNA and protein levels demonstrate its known short half and may be associated with cell cycle changes induced by HGF/SF [50,51].

## Bibliography

1. Graveel CR, DeGroot JD, Su Y, Koeman J, Dykema K, et al. (2009) Met induces diverse mammary carcinomas in mice and is associated with human basal breast cancer. Proc Natl Acad Sci U S A 106: 12909-12914.

2. Ponzo MG, Lesurf R, Petkiewicz S, O'Malley FP, Pinnaduwage D, et al. (2009) Met induces mammary tumors with diverse histologies and is associated with poor outcome and human basal breast cancer. Proc Natl Acad Sci U S A 106: 12903-12908.

3. Gastaldi S, Comoglio PM, Trusolino L (2010) The Met oncogene and basal-like breast cancer: another culprit to watch out for? Breast Cancer Res 12: 208.

4. Hochgrafe F, Zhang L, O'Toole SA, Browne BC, Pinese M, et al. (2010) Tyrosine phosphorylation profiling reveals the signaling network characteristics of Basal breast cancer cells. Cancer Res 70: 9391-9401.

5. Byers S, Park M, Sommers C, Seslar S (1994) Breast carcinoma: a collective disorder. Breast Cancer Res Treat 31: 203-215.

6. Tolgay Ocal I, Dolled-Filhart M, D'Aquila TG, Camp RL, Rimm DL (2003) Tissue microarray-based studies of patients with lymph node negative breast carcinoma show that met expression is associated with worse outcome but is not correlated with epidermal growth factor family receptors. Cancer 97: 1841-1848.

7. Camp RL, Rimm EB, Rimm DL (1999) Met expression is associated with poor outcome in patients with axillary lymph node negative breast carcinoma. Cancer 86: 2259-2265.

8. Dumont JE, Dremier S, Pirson I, Maenhaut C (2002) Cross signaling, cell specificity, and physiology. Am J Physiol Cell Physiol 283: C2-28.

9. van de Vijver MJ, He YD, van't Veer LJ, Dai H, Hart AA, et al. (2002) A gene-expression signature as a predictor of survival in breast cancer. N Engl J Med 347: 1999-2009.

10. Sawada Y, Tamada M, Dubin-Thaler BJ, Cherniavskaya O, Sakai R, et al. (2006) Force sensing by mechanical extension of the Src family kinase substrate p130Cas. Cell 127: 1015-1026.

11. Sridhar SC, Miranti CK (2006) Tetraspanin KAI1/CD82 suppresses invasion by inhibiting integrin-dependent crosstalk with c-Met receptor and Src kinases. Oncogene 25: 2367-2378.

12. Cowell LN, Graham JD, Bouton AH, Clarke CL, O'Neill GM (2006) Tamoxifen treatment promotes phosphorylation of the adhesion molecules, p130Cas/BCAR1, FAK and Src, via an adhesion-dependent pathway. Oncogene 25: 7597-7607.

13. Brinkman A, de Jong D, Tuinman S, Azaouagh N, van Agthoven T, et al. (2009) The substrate domain of BCAR1 is essential for anti-estrogen-resistant proliferation of human breast cancer cells. Breast Cancer Res Treat.

14. Frasor J, Chang EC, Komm B, Lin CY, Vega VB, et al. (2006) Gene expression preferentially regulated by tamoxifen in breast cancer cells and correlations with clinical outcome. Cancer Res 66: 7334-7340.

15. Yoneda-Kato N, Kato JY (2008) Shuttling imbalance of MLF1 results in p53 instability and increases susceptibility to oncogenic transformation. Mol Cell Biol 28: 422-434.

16. Minoshima Y, Hori T, Okada M, Kimura H, Haraguchi T, et al. (2005) The constitutive centromere component CENP-50 is required for recovery from spindle damage. Mol Cell Biol 25: 10315-10328.

17. Li Y, Zou L, Li Q, Haibe-Kains B, Tian R, et al. Amplification of LAPTM4B and YWHAZ contributes to chemotherapy resistance and recurrence of breast cancer. Nat Med 16: 214-218.

18. Garcia-Guzman M, Dolfi F, Russello M, Vuori K (1999) Cell adhesion regulates the interaction between the docking protein p130(Cas) and the 14-3-3 proteins. J Biol Chem 274: 5762-5768.

19. Lim R, Winteringham LN, Williams JH, McCulloch RK, Ingley E, et al. (2002) MADM, a novel adaptor protein that mediates phosphorylation of the 14-3-3 binding site of myeloid leukemia factor 1. J Biol Chem 277: 40997-41008.

20. Shen M, Yen A (2008) c-Cbl interacts with CD38 and promotes retinoic acid-induced differentiation and G0 arrest of human myeloblastic leukemia cells. Cancer Res 68: 8761-8769.

21. Wang L, Rudert WA, Loutaev I, Roginskaya V, Corey SJ (2002) Repression of c-Cbl leads to enhanced G-CSF Jak-STAT signaling without increased cell proliferation. Oncogene 21: 5346-5355.

22. Scheuermann RH, Racila E, Tucker T, Yefenof E, Street NE, et al. (1994) Lyn tyrosine kinase signals cell cycle arrest but not apoptosis in B-lineage lymphoma cells. Proc Natl Acad Sci U S A 91: 4048-4052.

23. Sprangers M, Feldhahn N, Herzog S, Hansmann ML, Reppel M, et al. (2006) The SRC family kinase LYN redirects B cell receptor signaling in human SLP65-deficient B cell lymphoma cells. Oncogene 25: 5056-5062.

24. Pathan NI, Geahlen RL, Harrison ML (1996) The protein-tyrosine kinase Lck associates with and is phosphorylated by Cdc2. J Biol Chem 271: 27517-27523.

25. Wu Y, Wang G, Wei J, Wen X (2005) Survivin protein expression positively correlated with proliferative activity of cancer cells in bladder cancer. Indian J Med Sci 59: 235-242.

26. Tanaka K, Iwamoto S, Gon G, Nohara T, Iwamoto M, et al. (2000) Expression of survivin and its relationship to loss of apoptosis in breast carcinomas. Clin Cancer Res 6: 127-134.

27. Nandi AK, Ford T, Fleksher D, Neuman B, Rapoport AP (2007) Attenuation of DNA damage checkpoint by PBK, a novel mitotic kinase, involves protein-protein interaction with tumor suppressor p53. Biochem Biophys Res Commun 358: 181-188.

28. Matsumoto S, Abe Y, Fujibuchi T, Takeuchi T, Kito K, et al. (2004) Characterization of a MAPKK-like protein kinase TOPK. Biochem Biophys Res Commun 325: 997-1004.

29. Buban T, Toth L, Tanyi M, Kappelmayer J, Antal-Szalmas P (2009) [Ki-67 -- new faces of an old player]. Orv Hetil 150: 1059-1070.

30. Koff A, Cross F, Fisher A, Schumacher J, Leguellec K, et al. (1991) Human cyclin E, a new cyclin that interacts with two members of the CDC2 gene family. Cell 66: 1217-1228.

31. Maxia C, Perra MT, Demurtas P, Minerba L, Murtas D, et al. (2008) Expression of survivin protein in pterygium and relationship with oxidative DNA damage. J Cell Mol Med 12: 2372-2380.

32. Jansen MP, Reijm EA, Sieuwerts AM, Ruigrok-Ritstier K, Look MP, et al. High miR-26a and low CDC2 levels associate with decreased EZH2 expression and with favorable outcome on tamoxifen in metastatic breast cancer. Breast Cancer Res Treat.

33. Scaltriti M, Eichhorn PJ, Cortes J, Prudkin L, Aura C, et al. Cyclin E amplification/overexpression is a mechanism of trastuzumab resistance in HER2+ breast cancer patients. Proc Natl Acad Sci U S A 108: 3761-3766.

34. Yamashita S, Masuda Y, Kurizaki T, Haga Y, Murayama T, et al. (2007) Survivin expression predicts early recurrence in early-stage breast cancer. Anticancer Res 27: 2803-2808.

35. Pise-Masison CA, Radonovich M, Dohoney K, Morris JC, O'Mahony D, et al. (2009) Gene expression profiling of ATL patients: compilation of disease-related genes and evidence for TCF4 involvement in BIRC5 gene expression and cell viability. Blood 113: 4016-4026.

36. Zhu F, Zykova TA, Kang BS, Wang Z, Ebeling MC, et al. (2007) Bidirectional signals transduced by TOPK-ERK interaction increase tumorigenesis of HCT116 colorectal cancer cells. Gastroenterology 133: 219-231.

37. Guo LL, Gao P, Wu YG, Jian WC, Hao CY, et al. (2007) Alteration of cyclin D1 in Chinese patients with breast carcinoma and its correlation with Ki-67, pRb, and p53. Arch Med Res 38: 846-852.

38. Truchet I, Jozan S, Baron S, Frongia C, Balaguer P, et al. (2008) Estrogen and antiestrogen-dependent regulation of breast cancer cell proliferation in multicellular spheroids: Influence of cell microenvironment. Int J Oncol 32: 1033-1039.

39. Colleoni M, Rotmensz N, Peruzzotti G, Maisonneuve P, Viale G, et al. (2004) Minimal and small size invasive breast cancer with no axillary lymph node involvement: the need for tailored adjuvant therapies. Ann Oncol 15: 1633-1639.

40. Shaye A, Sahin A, Hao Q, Hunt K, Keyomarsi K, et al. (2009) Cyclin E deregulation is an early event in the development of breast cancer. Breast Cancer Res Treat 115: 651-659.

41. Potemski P, Kusinska R, Pasz-Walczak G, Piekarski JH, Watala C, et al. (2009) Prognostic relevance of cyclin E expression in operable breast cancer. Med Sci Monit 15: MT34-40.

42. Agarwal R, Gonzalez-Angulo AM, Myhre S, Carey M, Lee JS, et al. (2009) Integrative analysis of cyclin protein levels identifies cyclin b1 as a classifier and predictor of outcomes in breast cancer. Clin Cancer Res 15: 3654-3662.

43. Decker T, Hipp S, Ringshausen I, Bogner C, Oelsner M, et al. (2003) Rapamycin-induced G1 arrest in cycling B-CLL cells is associated with reduced expression of cyclin D3, cyclin E, cyclin A, and survivin. Blood 101: 278-285.

44. Liu J, Campen A, Huang S, Peng SB, Ye X, et al. (2008) Identification of a gene signature in cell cycle pathway for breast cancer prognosis using gene expression profiling data. BMC Med Genomics 1: 39.

45. Taher TE, Tjin EP, Beuling EA, Borst J, Spaargaren M, et al. (2002) c-Cbl is involved in Met signaling in B cells and mediates hepatocyte growth factor-induced receptor ubiquitination. J Immunol 169: 3793-3800.

46. Gao CF, Vande Woude GF (2005) HGF/SF-Met signaling in tumor progression. Cell Res 15: 49-51.

47. Edakuni G, Sasatomi E, Satoh T, Tokunaga O, Miyazaki K (2001) Expression of the hepatocyte growth factor/c-Met pathway is increased at the cancer front in breast carcinoma. Pathol Int 51: 172-178.

48. Grigioni WF, Fiorentino M, D'Errico A, Ponzetto A, Crepaldi T, et al. (1995) Overexpression of c-met protooncogene product and raised Ki67 index in hepatocellular carcinomas with respect to benign liver conditions. Hepatology 21: 1543-1546.

49. Keyhanian K, Edalat R, Oghalaei A, Askary N, Golshani A, et al. (2007) Effect of hepatocyte growth factor (HGF) on the level of Survivin & XIAP expression in several human cancer cell lines, after treating with DNA damaging agent. Mol Cell Biochem 304: 199-205.

50. Zhao J, Tenev T, Martins LM, Downward J, Lemoine NR (2000) The ubiquitin-proteasome pathway regulates survivin degradation in a cell cycle-dependent manner. J Cell Sci 113 Pt 23: 4363-4371.

51. Beardmore VA, Ahonen LJ, Gorbsky GJ, Kallio MJ (2004) Survivin dynamics increases at centromeres during G2/M phase transition and is regulated by microtubule-attachment and Aurora B kinase activity. J Cell Sci 117: 4033-4042.
